# Supplementary material for: Pathway connectivity and signaling coordination in the yeast stress-activated signaling network
Source: Mol Syst Biol. 2014 Nov 19;10(11):759. doi: 10.15252/msb.20145120 (PMC4299600; doi:10.15252/msb.20145120)
Supplement: Supplementary file 1 — Supplementary Information [file msb0010-0759-sd1.pdf]

Supplementary Information for Chasman & Ho *et al.*,
"Coordination and interconnectivity in the inferred stress-activated signaling network from yeast"

Corresponding authors: Audrey Gasch (agasch@wisc.edu), Mark Craven (craven@biostat.wisc.edu)

Contents

Supplementary figures **2**

Supplementary tables **2**

S1 Computational methods **3**
- S1.1 Outline of the approach . . . 3
- S1.2 Data supplied to subnetwork inference method . . . 5
  - S1.2.1 Experimental data . . . 5
  - S1.2.2 Indirect interactions from domain knowledge . . . 6
  - S1.2.3 Background network . . . 6
  - S1.2.4 Candidate paths . . . 7

S2 Computational analysis **8**
- S2.1 Enrichment of consensus subnetwork with known relevant proteins . . . 9
- S2.2 Stability analysis . . . 10
- S2.3 Lesion testing . . . 11
- S2.4 Testing variations on candidate path length . . . 13
- S2.5 Testing the effect of reordering the objective function components . . . 14

S3 Analysis of newly implicated regulators **15**

S4 Cdc14 is a central regulator in the NaCl response **16**

S5 References **18**

S6 List of supplementary datasets **22**

Supplementary figures

S1 Signaling proteins required for acquisition of H_2O_2 resistance 23
S2 Graphical representation of affected genes 24
S3 Phospho-proteome network 25
S4 Overlap in targets of all interrogated regulators 27
S5 Defective *in vivo* CTD phosphorylation in *hog1*Δ cells responding to NaCl 28
S6 Cdc14 is a central regulator in the NaCl response 29
S7 Change in Rpb3 occupancy at ESR-gene promoters. 30
S8 Diagram of the procedure for optimization in the IP 31
S9 Example of ESR bifurcation score calculation 32
S10 Stability analysis results 33
S11 Precision-recall curves for the lesioned IPs 34
S12 Similarity of ensembles inferred by the lesioned IPs 35
S13 Testing variations on the length of candidate paths. 36
S14 Testing the reordering of objective function components. 37

Supplementary tables

S1 Validation of predicted regulators 38
S2 Provenance of background network 39
S3 Sets of network elements that are provided as input to the method. 41
S4 Integer program variables 41
S5 Coverage of each source's targets and candidate TF/RBPs by the candidate paths . 42
S6 Top 15 consensus nodes ranked by degree. 43
S7 Enrichment analysis results 44
S8 Enrichment analysis of lesioned IPs 45
S9 Enrichment analysis of subnetworks inferred from varied candidate path lengths . . 46
S10 Enrichment analysis of reordered IPs 47

S1 Computational methods

We developed an integer linear programming-based approach (IP, for short) to infer the pathways through which the signaling regulator mutants perturb the regulation of their affected downstream targets. Using a *background network* of directed and undirected intracellular interactions, the method infers a *subnetwork* that predicts the *paths* by which each interrogated *source* regulator is connected to its downstream *targets*, identified as dysregulated genes in the source mutant responding to NaCl treatment. Each path is a directed, linear chain of interactions between yeast gene products. The final interaction in each path represents the regulation of the target gene by a sequence-specific transcription factor or RNA-binding protein. The IP's objective function favors the inclusion of salt-responsive proteins, defined as those with differential phosphorylation upon NaCl treatment (phospho-proteomic hits) and those required for normal fitness during the NaCl response (fitness-contribution hits).

S1.1 Outline of the approach

In this section, we present a detailed description of the three steps of our inference method, illustrated in Figure 3.

Step 1: Gather the experimental data and a background network. Figure 3A shows the input to the method: data from experiments that interrogate the yeast salt stress response (as described in the manuscript), and a background network of intracellular interactions. The background network contains three types of nodes: proteins (shown as ellipses), TFs and RNA-binding proteins (diamonds), and genes/mRNAs (rectangles). The experimental data comes in the following forms:

- *Source-target pairs*, each consisting of one interrogated *source* regulator and one of its identified downstream gene/mRNA targets (sources are shown with bold outlines; targets are shown as rectangles)
- *Receptor-source pairs*, where necessary, which represent directed relationships gathered from the literature. For example, to capture upstream stress sensors, we included knowledge about two transmembrane receptors that are upstream of Hog1.
- *Fitness-contribution hits*, genes that result in defective stress resistance acquisition when they are knocked out (shown in blue in Figure 3)
- *Phospho-proteomic hits*, proteins that are differentially phosphorylated under salt stress conditions (shown in yellow in Figure 3)

We refer to the fitness-contribution hits and phospho-proteomic hits together as *hits*. The sets are not mutually exclusive.

The background network (described in Section S1.2.3) consists of a variety of types of biological interactions, namely protein-protein and protein-nucleic acid interactions, gathered from

public databases. It is represented as a graph in which the nodes represent either proteins or genes/mRNAs, and the edges represent interactions between them. If the direction of an interaction is known (as in a kinase-substrate interaction), then the corresponding edge is directed. Otherwise, the edge is undirected.

Step 2: Generate candidate paths to explain experimental observations. The inferred subnetwork must provide directed paths between each source and all of its targets, and so we first use the background network to enumerate all possible candidate paths. Each candidate source-target path must end with an interaction that represents regulation of the target mRNA: either the binding of a transcription factor (TF) to the target's gene, or the binding of an RNA-binding protein (RBP) to the target's mRNA. We also enumerate other kinds of candidate paths in order to capture connections among salt-responsive proteins that may be missed by cataloging only source-target paths. In Figure 3B, we show the three kinds of paths that we identify:

- *Source-target paths*, which connect interrogated sources to their targets
- *Receptor-source paths*, which offer paths between upstream stress sensors and downstream sources
- *Fitness-contribution hit-source* and *source-source paths*, which identify connections between potential regulators

The inferred subnetwork is a collection of directed paths that make a consistent assignment for the direction of each edge.

Step 3: Solve an IP to infer an ensemble of subnetworks. To perform inference, we construct an integer linear program (Figure 3C) whose constraints and objective functions describe a subnetwork that conforms to the following desiderata:

- Each source is connected to of all of its targets by directed paths
- Any provided upstream relationships (receptor-source pairs) are explained by directed paths
- Directed paths reveal connections between fitness-contribution hits and sources that are proximal in the background network
- Each edge is assigned only one direction
- Proteins with phospho-proteomic or fitness evidence are favored for inclusion in the subnetwork
- The subnetwork includes a minimal number of nodes that are not supported by experimental evidence

Because both the experimental data and the background network are incomplete, there will be many possible inferred subnetworks that explain the experimental data equally well. To quantify our confidence in the relevance of each protein and interaction, we infer an ensemble of optimal subnetworks. Confidence in a protein (interaction, path) is calculated as the fraction of the subnetworks in the ensemble that predict that the protein (interaction, path) is relevant.

Figure 3C shows two inferred relevant subnetworks for the given example. In each subnetwork, the predicted relevant edges (interactions) and nodes (proteins) are indicated with black outlines; rejected edges and nodes are in grey. A direction has been inferred for each formerly undirected interaction that is predicted to be relevant.

S1.2 Data supplied to subnetwork inference method

The inputs to the IP method are the data capturing the cell's response to salt stress (transcriptome changes, fitness-contributions, and phosphorylation responses), additional, known relationships between stress response factors curated from literature, and a background network consisting of publicly available yeast intracellular interactions.

S1.2.1 Experimental data

The primary goal of the approach is to provide explanations for the measured salt-specific transcriptomic changes. We also use two additional sources of salt-specific experimental data.

Source-target pairs. From the transcriptomic data measured in each of the original signaling mutants, we identified the set of downstream genes with dysregulated salt-responsive expression. We then extracted what we refer to as *source-target pairs*, each consisting of a single signaling protein (*source*) and a gene that was dysregulated in the mutant under salt stress (*target*). We divided the set of pairs into two categories, based on whether or not the source is a known transcription factor (TF). For non-TF sources, the effect on the targets is assumed to be indirect, and mediated by interactions between other gene products. For sources that are TFs, it is assumed that they affect their targets directly.

Fitness-contribution hits. Previously, Berry *et al.* (2011) identified yeast mutants that conferred a defect in acquired stress resistance after salt pretreatment. For each of these mutants, we applied the label *fitness-contribution hit* to the gene product represented in our background network, because of the mutation's negative effect on yeast fitness under salt stress.

Phospho-proteomic hits. We applied the label *phospho-proteomic hit* to each protein that showed differential phosphorylation under salt stress).

S1.2.2 Indirect interactions from domain knowledge

Our method can take advantage of domain knowledge about the salt stress response in order to provide a scaffold for the inferred subnetwork. Here, we wanted to capture the upper-most stress sensors that may otherwise be missed in connecting sources to their downstream targets. We identified well-known indirect relationships between two transmembrane receptors, Sln1 and Sho1, and one of the sources, Hog1 (Saito & Tatebayashi, 2004). We provided this information to our method as *receptor-source* pairs: (Sln1, Hog1) and (Sho1, Hog1). During inference, the method selects directed paths from the two receptors to Hog1.

S1.2.3 Background network

In constructing our background network, we identified a variety of binary interactions that are relevant to intracellular signaling and gene expression regulation. The background network represents protein-protein (including kinase-substrate), protein-DNA, and protein-RNA interactions.

Physical protein-protein interactions (PPIs) were sourced primarily from the BioGRID database (Stark *et al.*, 2006); these included both undirected PPIs and directed post-translational modifications, such as ubiquitination. To identify high-confidence interactions, we retrieved interactions that were supported by at least two separate experimental methods. In order to specifically capture signaling pathways, we supplemented the PPI network with additional high-confidence protein-protein interactions involving kinases and phosphatases (Breitkreutz *et al.*, 2010; Fasolo *et al.*, 2011; Sharifpoor *et al.*, 2011). Directed kinase-substrate and phosphatase-substrate interactions were identified from BioGRID, data published by Ptacek *et al.* (2005), and the KID database (Sharifpoor *et al.*, 2011).

Additionally, we extracted binary interactions from metabolic pathway data (Heavner *et al.*, 2012), which represent interactions between enzymes that catalyze adjacent metabolic reactions, and information about which proteins are components of annotated protein complexes (Heavner *et al.*, 2012; Pu *et al.*, 2009).

To represent the direct regulation of the target genes/mRNAs, we gathered protein-DNA (Abdulrehman *et al.*, 2011; Everett *et al.*, 2009; Guelzim *et al.*, 2002; MacIsaac *et al.*, 2006; Venters *et al.*, 2011) and protein-RNA (Hogan *et al.*, 2008; Scherrer *et al.*, 2010; Tsvetanova *et al.*, 2010) interactions from several publications. We supplemented these with two sources of protein-DNA interactions gathered under stress conditions (Huebert *et al.*, 2012; Ni *et al.*, 2009).

After manual inspection of the background network neighborhoods of the interrogated mutants, we added a set of 17 missing interactions between the mutants and nearby regulators based on known interactions in the literature.

While the types of biological interactions in the background network are rich and diverse, we use a simplified representation as input to the computational method. The background network is represented as a graph, in which nodes represent genes and gene products, and edges represent interactions. A gene may be represented as two separate nodes in the background network: one

representing the protein, and, for targets, one representing the DNA or mRNA. Each interaction may have a direction: for example, transcriptional regulatory interactions are directed, but most protein-protein interactions are not. (Protein-protein interactions representing post-translational modifications, such as phosphorylation, are directed.)

We represented each protein complex as an additional node, and added a directed edge to the complex from each of its component proteins. Using protein-protein interactions, we also inferred undirected interactions between protein complexes and between a complex and a single protein. If more than 50% of the possible protein-protein interactions between two protein complexes (or between a complex and a protein) were present in the data, then we added an undirected edge between the two complexes (or complex and protein).

Finally, we collapsed Dot6 and its paralog Tod6 into the same protein node in the background network, because they have been observed to function redundantly and because our measured targets were identified in a *dot6*Δ*tod6*Δ double-mutant strain. We accomplished this by replacing the two separate proteins with a new protein node, named Dot6/Tod6.

In total, the background network consisted of 5,130 nodes representing proteins or protein complexes and 6,481 nodes representing DNA or RNA. There were 29,936 unique, interacting pairs of proteins (27% having a known direction) and 260,365 unique pairs of one protein and one nucleic acid sequence. Information about the provenance of the background network is provided in Supplementary Table 2. The background network itself (minus transcriptional regulatory edges that do not involve experimental targets) is available in Supplementary Dataset S6.

S1.2.4 Candidate paths

An inferred subnetwork is composed of a union of directed paths through the background network, each path providing an explanation for pairs identified from experimental data or domain knowledge. As a set-up step before proceeding with inference, we must enumerate candidate directed paths for each type of salt-relevant pair by performing searches through the background network (Figure 3B). When we incorporate an undirected edge in a path, we record the direction of the edge that allows the path to move forward from the source to the target. The nodes and edges in the candidate paths are indicated in the Cytoscape file provided as Supplementary Dataset S6. Experiments varying the lengths of these paths are discussed in Section S2.4.

Candidate source-target paths. To account for the effect of a source mutant on its dysregulated targets, we must provide directed candidate paths that begin with the source and end with an interaction that represents the binding of a TF (or RBP) to the target gene (or mRNA). In this section we describe our process for enumerating these paths.

For the five sources that are themselves transcription factors (Dot6/Tod6, Msn2, Rim101, Swc3, and Swc5), we assumed that they bind their targets directly. For these sources, each source-target path consisted of only one interaction.

We enumerated longer paths to account for the sources that are not transcription factors. First,

we identified which TFs and RBPs present in the background network could plausibly account for the effect of each source on its targets. We considered a relationship between a source and a TF (or RBP) if (a) the binding data for the TF/RBP was gathered under salt stress conditions (Ni *et al.*, 2009) or (b) the set of genes bound by the TF/RBP were significantly overrepresented in the source's targets. Overrepresentation is decided by a p-value $<$ 0.05 given by a hypergeometric test of the overlap between the two sets. Because we were only assembling a candidate list of TFs for each source, we did not correct for multiple testing at this step.

After identifying candidate TFs and RBPs for each source, we enumerated possible directed paths between all source-target pairs through the background network, terminating with an interaction between one of the candidate TFs/RBPs and the target. Because the experimental data represents transcriptional changes that happen on a short time scale, the interactions in the path (except for the last) were limited to either interactions between proteins or between an RBP and the protein node for an mRNA that it binds. In other words, we did not allow connections from target genes/mRNAs to proteins, which would require both transcription and subsequent translation of the protein encoded by the target.

We applied a limit on the number of interactions in each path for the sake of tractability. For each source that was not a TF/RBP, we first enumerated all paths of up to three interactions. If by doing so we were unable to reach at least 50% of the source's candidate TFs/RBPs, we searched out until that goal percentage was reached, allowing up to five interactions total. By this process we were able to reach on average 78% of each source's targets and 75% of each source's candidate TFs/RBPs. Supplementary Table S5 shows the coverage of each source's targets and candidate TFs/RBPs by the candidate paths.

Candidate fitness-contribution hit-source and source-source paths. In addition to explaining the effects of the sources on their targets, we were also interested in identifying other connections among salt-responsive proteins that may not lie along source-target paths: among fitness-contribution hits and sources, and among the sources themselves. We enumerated short candidate paths (up to two interactions) between each fitness-contribution hit and each source, and between each pair of sources. None of the interactions in these paths could represent the binding of a TF to a gene. For each pair, we searched for paths that proceed in both directions.

Candidate paths to explain indirect relationships from domain knowledge (receptor-source paths). In this step, we enumerated directed paths of up to five interactions that could connect the two upstream receptors, Sho1 and Sln1, to Hog1. As we did for the hit-source and source-source paths, we only allowed protein-protein and RBP-protein interactions in these paths.

S2 Computational analysis

We used the ensemble of subnetworks inferred by the IP method to make predictions about different aspects of the salt-specific subnetwork. We inspected the ensemble from a different view for

each type of prediction. By applying a threshold on confidence values represented by the ensemble, we defined three high-confidence, or consensus, views:

- **Consensus nodes**, defined as the set of nodes with at least 75% confidence; they were used to make predictions about the identity of key players in the salt response
- **Consensus edges**, defined as the set of edges with at least 75% confidence; they were used to make predictions about direct interactions between consensus nodes
- **Consensus paths**, defined as the set of paths with at least 75% confidence; they were used to make predictions about indirect relationships between proteins and genes/mRNAs (such as, which proteins mediate the signal between a given source and its targets)

In this section we describe the methodology used for each computational result presented in the manuscript, as well as a supplementary analysis of the contributions of multiple components of the IP method. All three consensus views of the subnetwork are available in Cytoscape format in Supplementary Dataset S8. A list of the 15 highest-degree consensus nodes is provided in Supplementary Table S6.

S2.1 Enrichment of consensus subnetwork with known relevant proteins

Because our curated list of true positives is known to be incomplete, we additionally evaluated our consensus protein node set (excluding target genes/mRNAs) against several external sources of likely-relevant genes and interactions. We tested the enrichment of consensus proteins with two sets of genes that are related to cell signaling and stress: (a) kinases and phosphatases, and (b) proteins retrieved from SGD's YeastMine (Balakrishnan *et al.*, 2012; Cherry *et al.*, 2012) with the queries 'stress regulation' or 'nutrient regulation'. We also tested for enrichment with essential genes from SGD (Cherry *et al.*, 2012), and for genetic interactions from BioGRID (Stark *et al.*, 2006). Finally, we also tested the consensus subnetwork for overrepresentation of the true positives and underrepresentation of the likely negatives described in Methods and Figure 4B.

Using the hypergeometric test, enrichment was tested both relative to the candidate network (the network given by the set of candidate paths) and to the background network. To separate out the effect of experimental hits on the enrichment score, we excluded them from the consensus nodes and the external gene sets. After filtering, 160 consensus nodes, 736 candidate nodes, and 4703 background network nodes remained. With experimental hits removed, the external gene sets consisted of 147 kinases and phosphatases, 237 general stress proteins, 910 essential genes, 70 true positives, and 1416 likely negatives.

Additionally, we tested for enrichment of genetic interactions based on the assumption that genetic interactions are more likely to occur between functionally related genes. For this test, we did not omit experimental hits. We extracted 141,507 genetic interactions reported in BioGRID (Stark *et al.* (2006), downloaded February 2013) and assumed a total of 16 million possible interactions

among 5,700 yeast genes. Consensus subnetwork enrichment for genetic interactions was calculated using the number of pairs of consensus nodes that have a reported genetic interaction.

We also tested the consensus subnetwork against the permuted subnetworks described in Methods and Figure 4B. For each ensemble inferred from permuted data, we defined a consensus node set using a 75% confidence threshold, and, for each external gene/interaction set, measured the proportion of each consensus node set that was contained in the external set. For each external gene set, we calculated the p-value as the fraction of the 1,000 permuted node sets that had an equal or higher proportion than the 'true' consensus node set. (For the likely negatives, we counted the number of permuted consensus node sets with equal or lower representation.) We did not filter out hits for this experiment, as the hit sets were different for each permutation.

In Supplementary Table S7, we show the proportion of the consensus subnetwork, candidate network, background network, and permuted subnetworks that is represented in each external gene set, and the p-values for comparisons to the consensus subnetwork. These results are summarized in the main manuscript.

S2.2 Stability analysis

As it is practically infeasible to identify all optimal solutions to the IP, we specify the number of subnetworks in the ensemble as an input to our method. The question arises of how the ensemble's predictions change as more solutions are identified. In this analysis, we compared the predictions made by the ensemble of 10,000 solutions to two smaller ensembles of 100 and 1,000 solutions each. As shown in Supplementary Figure S10A, the precision-recall curves for each ensemble were approximately the same, although the recall of low-confidence predictions increased slightly as more solutions were gathered. The representation of general stress proteins, kinases and phosphatases, and genetic interactions was also approximately the same across the different ensemble sizes.

Additionally, we compared the different sizes of ensembles based on the Jaccard similarity between their predictions. We calculate the similarity between two ensembles E (10,000 solutions) and E' (100 or 1,000 solutions) as follows. Using the variable y_n (node relevance) as an example, $p^E(y_n = 1)$ is E's confidence that node n is relevant. $\mathcal{N}$ refers to the set of all nodes (proteins).

$$similarity(E, E') = \frac{\sum_{n \in \mathcal{N}} |p^E(y_n = 1) - p^{E'}(y_n = 1)|}{\sum_{n \in \mathcal{N}} p^E(y_n = 1) + p^{E'}(y_n = 1) - |p^E(y_n = 1) - p^{E'}(y_n = 1)|} \tag{1}$$

We compared the 10,000-solution ensemble to the two smaller ensembles based on four variable types: node relevance, edge relevance, edge direction, and path relevance. Sources, targets, and upstream receptors were omitted from the node stability calculations because their relevance variables were fixed. When calculating the similarity of edge directions, only edges that were undirected in the background network were counted.

For all comparisons, the similarity between the 10,000-solution ensemble and the smaller ensemble was very high ($\geq$ 90%). In Supplementary Figure S10B, we provide a visualization of the results of each comparison, showing the number of elements that were shared or unique to each ensemble.

All together, our stability analysis results demonstrate that the distribution of solutions captured by CPLEX is not changed significantly by the collection of more solutions.

S2.3 Lesion testing

Our approach combines multiple sources of experimental data using a multi-step optimization procedure. It is useful to investigate the contribution of each ingredient to the predictions made by the inferred subnetwork ensemble. To do so, we ran lesioned versions of the IP, in each of which one component of the optimization procedure or one experimental data source was held aside, and inferred an ensemble of solutions, which we refer to as a *lesioned ensemble*. We evaluated the lesioned ensembles using the same computational evaluation metrics that we applied to the ensemble inferred by the complete IP (the *complete ensemble*): namely, accuracy in predicting held-aside test cases, and enrichment of relevant, externally-derived gene and interaction sets. Experimental hits were omitted from the test cases and the relevant, external gene sets. As an additional evaluation, we compared the lesioned ensembles to the complete ensemble based on the similarity of their predictions, as in the stability analysis presented in Section 2.2.

Having observed that predictions were highly stable regardless of the size of the ensemble, we performed the lesion experiments using an ensemble size of 1,000. Correspondingly, we compared the lesioned ensembles to the complete ensemble with 1,000 solutions.

Supplementary Figure S11A-F show comparative precision-recall curves for each lesioned ensemble. In Supplementary Table S8, we show the results of the enrichment analyses, including the proportion of each lesioned consensus subnetwork that is contained in each external gene and interaction set. For each relevant set, we compared its representation in the lesioned consensus to the complete consensus using a two-tailed z-test of proportions.

We discuss the contribution of each lesioned component:

Maximizing the number of connections between hits and sources (Supplementary Figure S11A). Removing this step alone slightly increased the predictive accuracy of the inferred ensemble, but did not significantly change the proportions of the consensus nodes that were represented by any relevant gene sets. We suggest three possible explanations for the change in precision: (a) the experimental hit set is noisy or includes proteins that are required for stress survival but not for signaling *per se*, (b) the set of test cases is relatively small and based on focused laboratory experimentation, and is thus likely to be incomplete, and (c) removing this objective function component left the resulting IP less constrained, allowing greater variation between the different solutions in the ensemble and more stratification between high-confidence predictions. There was also a small but statistically significant change in the proportion of genetic interactions. Despite the

small change in precision that it confers, including this step assists in the interpretation of the experimental data, as it reveals proximal connections between fitness-contribution hits and sources that are not captured by source-target paths.

Maximizing the number of experimental hits (Supplementary Figure S11B). Even though the hit nodes themselves were not considered in the accuracy analysis, maximizing their inclusion results in more accurate choices among the other nodes: the lesioned IP had a slightly lower PR curve compared to the complete IP. The representation of relevant gene sets did not change significantly; however, the lesioned consensus had a higher proportion of genetic interactions than the complete consensus node set.

The combined contribution of maximizing connections and hits (Supplementary Figure S11C). These two objective components are partially redundant, as maximizing the number of connections indirectly also maximizes the inclusion of hits. Therefore, we assessed the effect of removing both components. This resulted in a slight increase in precision. Additionally, while the lesioned consensus node set did not differ in the representation of relevant gene sets, it did contain a significantly higher proportion of genetically interacting pairs of nodes. The next lesion we performed attempted to tease apart the contributions of the two experimental hit sets.

Maximizing the inclusion of either of the two hit sets (Supplementary Figure S11D). To assess the hit sets separately, we performed two lesions, separately holding aside the fitness-contribution hits and the phospho-proteomic hits. The inclusion of phospho-proteomic hits in the IP appears to be useful for identifying higher precision subnetworks, demonstrating that some of the information contained in this hit set is congruent with current knowledge about the salt stress response. Conversely, holding aside the entire set of fitness-contribution hits (modulo the sources that are fitness-contribution hits) resulted in a moderate increase in precision, suggesting that this set of hits is noisy or partially disjoint to existing knowledge, and was responsible for the increased precision observed when both types of hits are held aside. Neither lesioned consensus node set shows a significant change in the representation of relevant node sets, though the lesioning of phospho-proteomic hits results in a statistically significant increase in genetic interactions. It is notable that most genetic interactions have been measured under standard growth conditions; it is likely that NaCl-specific genetic interactions are not represented in the test set.

Even though the predictive accuracy dropped slightly when fitness-contribution hits were maximized, our results may actually represent novel discoveries, as the set of true positives is heavily biased toward HOG pathways proteins. Regardless, we are willing to tolerate a slight drop in accuracy in exchange for increased interpretability. We believe that including the fitness-contribution hits in the IP is still useful; after all, part of the purpose of this method is to aid in the interpretation of the experimental hits. The resulting subnetwork reveals connections between the fitness-contributions and other components of the salt stress response, which may inspire further experimentation.

Minimizing the total number of nodes (Supplementary Figure S11E). Removing this step resulted in decreased precision and enrichment across the board, demonstrating that minimizing nodes is useful for reducing false positives.

Connecting receptor-source pairs (Supplementary Figure S11F). Including the receptor-source pairs appeared to significantly increase recall, but did not appear to have a great effect on precision or enrichment with relevant gene sets or genetic interactions. We believe that including these pairs makes the inferred subnetwork more interpretable by showing connections to well-understood interactions in the stress response.

Similar to our evaluation of the stability of the complete IP across different ensemble sizes, we measured the similarity between the complete and lesioned ensembles. A visualization of the differences and intersections between the ensembles' predictions is shown in Supplementary Figure S12. Except when nodes were not minimized, the lesioned ensembles contained fewer nodes, edges, and paths, and were mostly subsets of the complete ensemble. (In the figure, we observe that the size of the left-hand portion of each bar generally dominates the right-hand portion.) When nodes were not minimized, the reverse is observed. Path relevance and edge direction showed the most variablility, demonstrated by the longer right-hand ends to those bars.

S2.4 Testing variations on candidate path length

To supplement the lesion tests, we measured the effect of increasing and decreasing the maximum length of the candidate source-target and hit-source paths by one interaction. For each variation, we inferred an ensemble of 1,000 subnetworks, and compared the results to the original consensus ensemble on the basis of precision-recall and stability analyses (Supplementary Figure S13), as well as enrichment analysis on the nodes with $\geq$ 75% confidence (Supplementary Table S9). Because the candidate path sets are different between the original, complete IP and the new variations, we do not measure the stability of paths for these comparisons, and only compare node and edge relevance and edge direction.

Source-target paths (Supplementary Figure S13A-B). In the original IP, we enumerated paths of up to five interactions, stopping search early at the depth at which at least 50% of candidate TFs/RBPs for a given source were reached. We tested the effect of this early-stopping option by inferring subnetworks using candidate paths enumerated at three different lengths: three, four, or five interactions for all sources. At a path length of five, we were unable to complete the IP solution portion of the method due to the large number of paths generated by two sources, Hog1 and Mck1. As a compromise, we truncated the paths for those two sources to four, but searched for paths of length five for all other sources.

In general, the precision-recall curves and most enrichment scores do not appear to be very sensitive to the path length in the range tested; however, we see some patterns. The ensemble

inferred from candidate paths of length four appears to be nearly identical to the original IP under all measures, which is unsurprising, as search stopped at that length for most sources in the original setting. Stopping all search at length three results in a slight increase in precision, a slight decrease in recall, and a significant increase in genetic interactions. Stopping all search at length five results in a slight decrease in precision and a significant decrease in genetic interactions. As shown by the relative lengths of the ends of the bars in the stability analysis figure (Supplementary Figure S13B), increasing candidate path length also results in increased size of inferred subnetworks. The node content of the inferred ensembles is fairly similar, with more variation shown in edge relevance and direction.

Hit-source and source-source paths (Supplementary Figure S13C-D). In the original IP, we sought candidate paths consisting of up to two edges between the fitness-contribution hits and the tested sources. To assess the sensitivity of the method to this path length, we tested two alternatives: allowing only direct interactions (no intermediate nodes), and allowing longer paths of up to three edges (two intermediate nodes).

The shorter path length confers increased precision in the precision-recall curve; however, the 75%-confidence nodes from this subnetwork do not have statistically significantly different proportions of the gene sets tested for enrichment. There is a small but statistically significant increase in genetic interactions in the ensemble with shorter paths. This subnetwork includes 49 fitness-contribution hits, in contrast to the 106 included in the original consensus subnetwork, and is somewhat smaller.

The longer path length results in a large decrease in precision, weakly significant decreases in enrichment of true positives and genetic interactions, and a corresponding weakly significant increase in essential gene enrichment and likely negatives. This subnetwork includes 151 fitness-contribution hits and is much larger overall.

Considering these results, it appears that limiting the length of candidate hit-source and source-source paths is beneficial: longer paths result in larger subnetworks with decreased precision and decreased enrichment with other relevant gene and interaction sets. The preferred stringency of this limit may depend on how the inferred subnetwork will be used to guide further experiments. The shortest length path provides an increase in accuracy at the expense of not being able to provide connections to as many fitness-contribution hits.

S2.5 Testing the effect of reordering the objective function components

Our four objective functions can be summarized as representing two concepts: explain a *maximal* amount of the experimental data using a *minimal* subnetwork. To assess the sensitivity of the subnetwork inference method to the ordering of these concepts, we performed an experiment in which we reversed the order. The altered sequence of objective functions proceeds as follows, and can be compared to the sequence shown in Supplementary Figure S8:

1. Minimize nodes (one solution)

2. Maximize connections (one solution)
3. Maximize fitness-contribution and phospho-proteomic hits (one thousand solutions)
4. For each previous solution, maximize paths (one solution each)

Using this ordering of the objective functions, we inferred an ensemble of 1,000 subnetworks, and compared the results to the original consensus ensemble using precision-recall and stability analysis (Supplementary Figure S14), and enrichment analysis (Supplementary Table S10). Compared to the original IP, the IP with reversed objective function components (which we name “min-then-max”) achieves lower recall of the known regulators, no significantly different proportions of relevant gene sets, and a significant increase in genetic interaction enrichment. The resulting subnetworks are also overall smaller, as shown by the relative lengths of the colored portions of the bars in the stability analysis chart in Supplementary Figure S14B.

S3 Analysis of newly implicated regulators

The array results from the validation analysis provide new insights into the newly identified NaCl-responsive regulators. Several mutants had defects in ESR regulation. Measured targets of the Pho85 kinase, the Arf3 RBP, and CK2 subunit Cka2 were enriched for genes induced in the iESR. Cells lacking the cyclin-dependent Pho85 kinase also had a defect in repressing RP genes but displayed amplified induction of genes involved in respiration. Pho85 is thought of as a nutrient and cell cycle regulator but was also previously implicated in suppressing the ESR (Carroll *et al.*, 2001; Carroll & O’Shea, 2002; Moffat *et al.*, 2000). The *pho85*Δ mutant indeed had an apparent derepression of the ESR before stress (however this is hard to distinguish from ESR activation due to inherent stress in the sickly mutant cells). Cells lacking the Yak1 kinase had a defect in repressing RP genes in response to NaCl, likely due to the known Yak1-dependent regulation of RP regulators Ifh1 and/or Crf1 (Martin *et al.*, 2004; Kim *et al.*, 2011). Surprisingly, cells lacking Bck1 — a key MAPKKK in the cell wall integrity pathway — also displayed a defect in repressing RP genes upon NaCl treatment. The subnetwork inference predicts that Bck1 lies upstream of Yak1, based on the observation that Yak1 is an *in vitro* substrate of Bck1 (Ptacek *et al.*, 2005). As several studies have uncovered genetic interactions between these kinases (Lavina *et al.*, 2013; Cardona *et al.*, 2012; Hermansyah *et al.*, 2010), our results suggest that Bck1 is a previously unrealized regulator of Yak1 in the NaCl response.

Another example is the CK2 kinase complex, which regulates the iESR repressor Nrg1 and is known to affect stress-specific splicing and abundance of ribosome-related transcripts (Berkey & Carlson, 2006; Rudra *et al.*, 2007; Bergkessel *et al.*, 2011). We found that cells lacking either catalytic subunit (*CKA1* or *CKA2*) or both regulatory subunits (*CKB1/CKB2*) displayed a defective NaCl response, but that the defect differed for each mutant: *cka2*Δ cells displayed a defect in iESR gene induction, whereas the *cka1*Δ and *ckb1*Δ*ckb2*Δ mutants instead produced amplified repression of rESR genes. For both Cdc14 and CK2 subunits, the gene targets predicted

by the subnetwork significantly overlapped the measured targets, and 70-80% of scorable nodes predicted in their paths were supported by our analysis (see Supplementary Table S1).

We also found defective NaCl-provoked expression in two mutants not previously linked to stress. Kin2 is a poorly characterized serine/threonine kinase in yeast that is linked to exocytosis (Gasser *et al.*, 2007; Elbert *et al.*, 2005). Genes that displayed defective expression either basally or upon NaCl treatment were enriched for nutrient transporters, nutrient responsive regulators, and genes whose deletion produces morphology defects. Genes with amplified NaCl-responsive induction in the *kin2*Δ mutant were also enriched for targets of the meiosis regulator and transcriptional repressor, Sum1. These results are consistent with nutrient response and effects at the cellular periphery, where Kin2 is localized (Tibbetts *et al.*, 1994). Little is known about the kinase Nnk1, except from large-scale studies that show an interaction with Tor1 (Breitkreutz *et al.*, 2010). Most of the affected genes displayed amplified expression changes compared to wild type cells. There was little functional enrichment among the genes, aside of mobile-intron containing mitochondrial genes and genes regulated by TATA-dependent transcription.

S4 Cdc14 is a central regulator in the NaCl response

Cdc14 phosphatase is a key regulator of mitotic exit and acts in part as a Cdc28/Cdk1 antagonist, by reversing phosphorylation of Cdc28 targets (Visintin *et al.*, 1998). Cdc14 is sequestered in the nucleolus during most of the cell cycle but is released and distributed throughout the cell at anaphase (Visintin *et al.*, 1998; de Almeida *et al.*, 1999). In addition to triggering mitotic exist, Cdc14 also helps to set up subsequent cell-cycle phases, for example by priming Swi6 for nuclear import in G1 (Geymonat *et al.*, 2004) and licensing origins of replication for proper replication timing in S-phase (Zhai *et al.*, 2010; Dulev *et al.*, 2009; Bloom & Cross, 2007).

Here we show that Cdc14 is critical for mounting a normal NaCl response. Cells harboring the temperature sensitive *cdc14-3* allele had a major defect in several aspects of the NaCl response that could not be explained by cell-cycle defects. For these experiments, the mutant and its isogenic wild type were grown to log phase at 25°C then shifted to the nonpermissive 35°C for 90 minutes, leading to near-complete M-phase arrest of *cdc14-3* cells. At this time cells were exposed to 0.7M NaCl and followed for up to 2 hours. Notably, wild-type cells had a near normal NaCl response across the transcriptome under these conditions, with slightly smaller fold-changes compared to cells growing constitutively at 30°C. Thus the prior heat treatment had little effect on the wild-type response (as long as cells had 90 min to acclimate to the higher temperature, not shown). Mutant *cdc14-3* cells showed normal viability after heat shock (as scored by live-dead staining), but were more sensitive to short-term NaCl than wild-type cells (80% viability compared to 92% viability for wild-type).

To ensure that the apparent defect in NaCl response was not obscured by unique *cdc14-3* gene expression differences due to heat pretreatment, we compared expression in heat-treated *cdc14-3* and wild-type cells after the 90 min heat treatment but before addition of salt. Heat inactivation of

cdc14-3 caused only subtle differences in gene expression for most affected genes (Supplementary Figure S6A). The exception was cell cycle-regulated genes, whose expression reflected the M-phase arrest of the mutant (which produces reduced abundance of transcripts that peak in other cell-cycle phases, Figure 6F). The *cdc14-3* mutant had a slight induction of many stress-induced genes after heat inactivation but before NaCl addition. However, this cannot explain the defect in NaCl response of the mutant. First, many genes showed defective NaCl-responsive induction in the *cdc14-3* mutant despite no difference in pre-NaCl expression levels. This was also true for most NaCl-repressed genes, which showed little significant difference before NaCl addition in the *cdc14-3* mutant. Second, there were many genes for which pre-NaCl levels were slightly elevated in the inactivated *cdc14-3* mutant yet the fold-change in expression upon NaCl treatment happened at wild-type levels. Thus, expression differences in the *cdc14-3* mutant cannot be simply explained by highly aberrant transcript differences before NaCl addition.

In further support of a specific role for Cdc14 in the NaCl response, we found *cdc14-3* mutant cells growing at the non-permissive temperature had a partial defect in Hog1 localization (see main text). This was not due to M-phase arrest, since wild-type cells in G2/M phase showed robust Hog1 nuclear localization (Supplementary Figure S6B); furthermore, we found no defect in nuclear Hog1 accumulation in wild type cells arrested with nocodozole (not shown).

These results strongly suggest that the role of Cdc14 in the NaCl response is due to specific activation of the phosphatase, rather than an indirect effect of cell-cycle arrest. The NaCl-enhanced interaction between Cdc14 and Cka2 supports this conclusion. Because the mitotic function of Cdc14 is regulated via localization changes, we looked for nucleolar release of Cdc14 upon salt treatment. There was no gross relocalization of Cdc14-GFP after NaCl treatment (Supplementary Figure S6C); however, we cannot exclude a partial release of the phosphatase. Interestingly, a prior study found that nucleolar release of Cdc14 at anaphase is in part triggered by the Hog1 MAPKK Pbs2 (Reiser *et al.*, 2006); this observation bolsters our subnetwork's prediction that Pbs2 regulates Cdc14 activity upon NaCl treatment as well. Notably, we detected no interaction between immunoprecipitated Hog1 and Cdc14, either with or without prior cellular NaCl treatment (not shown).

Cdc14 is a known CTD phosphatase in yeast (Clemente-Blanco *et al.*, 2011) and humans (Guillamot *et al.*, 2011), prompting us to investigate whether it was required for the immediate drop in Ser2 and Ser5 phosphorylation upon NaCl treatment (see Figures 5E and Supplementary Figure S5). However, we found no obvious defect in the *cdc14-3* mutant responding to salt, relative to Pol II subunit Rpb3 (Supplementary Figure S6D). We noticed in several replicates that Rbp3 (normalized to a second internal control, Pab1) showed decreasing levels over time in the *cdc14-3* strain responding to NaCl at the non-permissive temperature, but not at 25°C (not shown). However, this was not always the case, raising question to its significance. It is intriguing, however, that the subnetwork connects Cdc14 to Rpb3 through the ubiquitin ligase Rsp5, which is known to control stability of polymerase subunits in response to other stresses (Beaudenon *et al.*, 1999). Future work will be required to dissect this interplay.

S5 References

Abdulrehman D, Monteiro PT, Teixeira MC, Mira NP, Lourenço AB, dos Santos SC, Cabrito TR, Francisco AP, Madeira SC, Aires RS, Oliveira AL, Sá-Correia I, Freitas AT (2011) YEASTRACT: providing a programmatic access to curated transcriptional regulatory associations in *Saccharomyces cerevisiae* through a web services interface. *Nucleic Acids Research* **39**: D136–D140

Alejandro-Osorio AL, Huebert DJ, Porcaro DT, Sonntag ME, Nillasithanukroh S, Will JL, Gasch AP (2009) The histone deacetylase Rpd3p is required for transient changes in genomic expression in response to stress. *Genome Biology* **10**: R57

Balakrishnan R, Park J, Karra K, Hitz BC, Binkley G, Hong EL, Sullivan J, Micklem G, Cherry JM (2012) YeastMine–an integrated data warehouse for *Saccharomyces cerevisiae* data as a multipurpose tool-kit. *Database (Oxford)* **2012**: bar062

Beaudenon SL, Huacani MR, Wang G, McDonnell DP, Huibregtse JM (1999) Rsp5 ubiquitin-protein ligase mediates DNA damage-induced degradation of the large subunit of RNA polymerase II in *Saccharomyces cerevisiae*. *Molecular and Cellular Biology* **19**: 6972–9

Bergkessel M, Whitworth GB, Guthrie C (2011) Diverse environmental stresses elicit distinct responses at the level of pre-mRNA processing in yeast. *RNA* **17**: 1461–1478

Berkey CD, Carlson M (2006) A specific catalytic subunit isoform of protein kinase CK2 is required for phosphorylation of the repressor Nrg1 in *Saccharomyces cerevisiae*. *Current Genetics* **50**(1): 1–10

Berry DB, Guan Q, Hose J, Haroon S, Gebbia M, Heisler LE, Nislow C, Giaever G, Gasch A (2011) Multiple means to the same end: the genetic basis of acquired stress resistance in yeast. *PLoS Genetics* **7**: e1002353

Bodenmiller B, Mueller LN, Mueller M, Domon B, Aebersold R (2007) Reproducible isolation of distinct, overlapping segments of the phosphoproteome. *Nature Methods* **4**: 231–237

Bloom J, Cross FR (2007) Novel role for Cdc14 sequestration: Cdc14 dephosphorylates factors that promote DNA replication. *Molecular and Cellular Biology* **27**: 842–53

Breitkreutz A, Choi H, Sharom JR, Boucher L, Neduva V, Larsen B, Lin ZY, Breitkreutz BJ, Stark C, Liu G, Ahn J, Dewar-Darch D, Reguly T, Tang X, Almeida R, Qin ZS, Pawson T, Gingras AC, Nesvizhskii AI, Tyers M (2010) A global protein kinase and phosphatase interaction network in yeast. *Science* **328**: 1043–1046

Cardona F, Del Olmo ML, Aranda A (2012) Phylogenetic origin and transcriptional regulation at the post-diauxic phase of SPI1, in *Saccharomyces cerevisiae*. *Cellular and Molecular Biology Letters* **17**: 393–407

Carroll AS, Bishop AC, DeRisi JL, Shokat KM, O'Shea EK (2001) Chemical inhibition of the Pho85 cyclin-dependent kinase reveals a role in the environmental stress response. *Proceedings of the National Academy of Science USA* **98**: 12578–83

Carroll AS, O'Shea EK (2002) Pho85 and signaling environmental conditions. *Trends in Biochemical Science* **27**: 87–93

Cherry JM, Hong EL, Amundsen C, Balakrishnan R, Binkley G, Chan ET, Christie KR, Costanzo MC, Dwight SS, Engel SR, Fisk DG, Hirschman JE, Hitz BC, Karra K, Krieger CJ, Miyasato SR, Nash RS, Park J, Skrzypek MS, Simison M, *et al.* (2012) Saccharomyces Genome Database: the genomics resource of budding yeast. *Nucleic Acids Research* **40**: D700–D705

Clemente-Blanco A, Sen N, Mayan-Santos M, Sacristan MP, Graham B, Jarmuz A, Giess A, Webb E, Game L, Eick D, Bueno A, Merkenschlager M, Aragon L (2011) Cdc14 phosphatase promotes segregation of telomeres through repression of RNA polymerase II transcription. *Nature Cell Biology* **13**: 1450–6

de Almeida A, Raccurt I, Peyrol S, Charbonneau M (1999) The *Saccharomyces cerevisiae* Cdc14 phosphatase is implicated in the structural organization of the nucleolus. *Biology of the Cell* **91**: 649–63

Dulev S, de Renty C, Mehta R, Minkov I, Schwob E, Strunnikov A (2009) Essential global role of CDC14 in DNA synthesis revealed by chromosome underreplication unrecognized by checkpoints in cdc14 mutants. *Proceedings of the National Academy of Science USA* **106**: 14466–71

Elbert M, Rossi G, Brennwald P (2005) The yeast Par-1 homologs Kin1 and Kin2 show genetic and physical interactions with components of the exocytic machinery. *Molecular Biology of the Cell* **16**: 532–49

Everett L, Vo A, Hannenhalli S (2009) PTM-Switchboard–a database of posttranslational modifications of transcription factors, the mediating enzymes and target genes. *Nucleic Acids Research* **37**

Fasolo J, Sboner A, Sun MGF, Yu H, Chen R, Sharon D, Kim PM, Gerstein M, Snyder M (2011) Diverse protein kinase interactions identified by protein microarrays reveal novel connections between cellular processes. *Genes & Development* **25**: 767–778

Forbes SA, Bindal N, Bamford S, Cole C, Kok CY, Beare D, Jia M, Shepherd R, Leung K, Menzies A, Teague JW, Campbell PJ, Stratton MR, Futreal PA (2011) COSMIC: mining complete cancer genomes in the Catalogue of Somatic Mutations in Cancer. *Nucleic Acids Research* **39**: D945–50

Gasser B, Sauer M, Maurer M, Stadlmayr G, Mattanovich D (2007) Transcriptomics-based identification of novel factors enhancing heterologous protein secretion in yeasts. *Applied and Environmental Microbiology* **73**: 6499–507

Geymonat M, Spanos A, Wells GP, Smerdon SJ, Sedgwick SG (2004) Clb6/Cdc28 and Cdc14 regulate phosphorylation status and cellular localization of Swi6. *Molecular and Cellular Biology* **24**: 2277–85

Guelzim N, Bottani S, Bourgine P, Képès F (2002) Topological and causal structure of the yeast transcriptional regulatory network. *Nature Genetics* **31**: 60–63

Gitter A, Carmi M, Barkai N, Bar-Joseph Z (2013) Linking the signaling cascades and dynamic regulatory networks controlling stress responses. *Genome Research* **23**: 365–376

Guillamot M, Manchado E, Chiesa M, Gomez-Lopez G, Pisano DG, Sacristan MP, Malumbres M (2011) Cdc14b regulates mammalian RNA polymerase II and represses cell cycle transcription. *Scientific Reports* **1**: 189

Hamosh A, Scott AF, Amberger JS, Bocchini CA, McKusick VA (2005) Online Mendelian Inheritance in Man (OMIM), a knowledgebase of human genes and genetic disorders. *Nucleic Acids Research* **33**: D514–7

Heavner BD, Smallbone K, Barker B, Mendes P, Walker LP (2012) Yeast 5 - an expanded reconstruction of the *Saccharomyces cerevisiae* metabolic network. *BMC Systems Biology* **6**: 55

Hermansyah, Lavina WA, Sugiyama M, Kaneko Y, Harashima S (2010) Identification of protein kinase disruptions as suppressors of the calcium sensitivity of *S. cerevisiae* Δ*ptp2*Δ*msg5* protein phosphatase double disruptant. *Archives of Microbiology* **192**: 157–65

Hogan DJ, Riordan DP, Gerber AP, Herschlag D, Brown PO (2008) Diverse RNA-binding proteins interact with functionally related sets of RNAs, suggesting an extensive regulatory system. *PLoS Biology* **6**: e255

Huebert DJ, Kuan PF, Keleş S, Gasch AP (2012) Dynamic changes in nucleosome occupancy are not predictive of gene expression dynamics but are linked to transcription and chromatin regulators. *Molecular and Cellular Biology* **32**: 1645–1653

Kim EM, Kim J, Kim YG, Lee P, Shin DS, Kim M, Hahn JS, Lee YS, Kim BG (2011) Development of high-throughput phosphorylation profiling method for identification of Ser/Thr kinase specificity. *Journal of Peptide Science* **17**: 392–7

Lavina WA, Shahsavarani H, Saidi A, Sugiyama M, Kaneko Y, Harashima S (2013) Suppression mechanism of the calcium sensitivity in *Saccharomyces cerevisiae ptp2*Δ*msg5*Δ double disruptant involves a novel HOG-independent function of Ssk2, transcription factor Msn2 and the protein kinase A component Bcy1. *Journal of Bioscience and Bioengineering*

MacIsaac K, Wang T, Gordon DB, Gifford D, Stormo G, Fraenkel E (2006) An improved map of conserved regulatory sites for *Saccharomyces cerevisiae*. *BMC Bioinformatics* **7**: 113+

Martin DE, Soulard A, Hall MN (2004) TOR regulates ribosomal protein gene expression via PKA and the Forkhead transcription factor FHL1. *Cell* **119**: 969–79

Moffat J, Huang D, Andrews B (2000) Functions of Pho85 cyclin-dependent kinases in budding yeast. *Progress in Cell Cycle Research* **4**: 97–106

Ni L, Bruce C, Hart C, Leigh-Bell J, Gelperin D, Umansky L, Gerstein MB, Snyder M (2009) Dynamic and complex transcription factor binding during an inducible response in yeast. *Genes & Development* **23**: 1351–1363

Ptacek J, Devgan G, Michaud G, Zhu H, Zhu X, Fasolo J, Guo H, Jona G, Breitkreutz A, Sopko R, McCartney RR, Schmidt MC, Rachidi N, Lee SJ, Mah AS, Meng L, Stark MJR, Stern DF, Virgilio CD, Tyers M, *et al.* (2005) Global analysis of protein phosphorylation in yeast. *Nature* **438**: 679–684

Pu S, Wong J, Turner B, Cho E, Wodak SJ (2009) Up-to-date catalogues of yeast protein complexes. *Nucleic Acids Research* **37**: 825–831

Reiser V, D'Aquino KE, Ee LS, Amon A (2006) The stress-activated mitogen-activated protein kinase signaling cascade promotes exit from mitosis. *Molecular Biology of the Cell* **17**: 3136–46

Rudra D, Mallick J, Zhao Y, Warner JR (2007) Potential interface between ribosomal protein production and pre-rRNA processing. *Molecular and Cellular Biology* **27**: 4815–4824

Saito H, Tatebayashi K (2004) Regulation of the osmoregulatory HOG MAPK cascade in yeast. *Journal of Biochemistry* **136**: 267–272

Scherrer T, Mittal N, Janga SC, Gerber AP (2010) A screen for RNA-binding proteins in yeast indicates dual functions for many enzymes. *PLoS One* **5**: e15499

Sharifpoor S, Ba ANN, Young JY, van Dyk D, Friesen H, Douglas AC, Kurat CF, Chong YT, Founk K, Moses AM, Andrews BJ (2011) A quantitative literature-curated gold standard for kinase-substrate pairs. *Genome Biology* **12**: R39

Soufi B, Kelstrup CD, Stoehr G, Frölich F, Walther TC, Olsen JV (2009) Global analysis of the yeast osmotic stress response by quantitative proteomics. *Molecular BioSystems* **5**: 1337–1346

Spellman PT, Sherlock G, Zhang MQ, Iyer VR, Anders K, Eisen MB, Brown PO, Botstein D, Futcher B (1998) Comprehensive identification of cell cycle-regulated genes of the yeast *Saccharomyces cerevisiae* by microarray hybridization. *Molecular Biology of the Cell* **9**: 3273–97

Stark C, Breitkreutz BJJ, Reguly T, Boucher L, Breitkreutz A, Tyers M (2006) BioGRID: a general repository for interaction datasets. *Nucleic Acids Research* **34**

Tibbetts M, Donovan M, Roe S, Stiltner AM, Hammond CI (1994) KIN1 and KIN2 protein kinases localize to the cytoplasmic face of the yeast plasma membrane. *Experimental Cell Research* **213**: 93–9

Tsvetanova NG, Klass DM, Salzman J, Brown PO (2010) Proteome-wide search reveals unexpected RNA-binding proteins in *Saccharomyces cerevisiae*. *PLoS One* **5**

Venters BJ, Wachi S, Mavrich TN, Andersen BE, Jena P, Sinnamon AJ, Jain P, Rolleri NS, Jiang C, Hemeryck-Walsh C, Pugh BF (2011) A comprehensive genomic binding map of gene and chromatin regulatory proteins in *Saccharomyces*. *Molecular Cell* **41**: 480–492

Visintin R, Craig K, Hwang ES, Prinz S, Tyers M, Amon A (1998) The phosphatase Cdc14 triggers mitotic exit by reversal of Cdk-dependent phosphorylation. *Molecular Cell* **2**: 709–18

Woods JO, Singh-Blom UM, Laurent JM, McGary KL, Marcotte EM (2013) Prediction of gene-phenotype associations in humans, mice, and plants using phenologs. *BMC Bioinformatics* **14**: 203

Zhai Y, Yung PY, Huo L, Liang C (2010) Cdc14p resets the competency of replication licensing by dephosphorylating multiple initiation proteins during mitotic exit in budding yeast. *Journal of Cell Science* **123**: 3933–43

S6 List of supplementary datasets

S1 Microarray data

S2 Finalized list of targets

S3 Average fold-changes of phospho sites

S4 Human orthologs from the (A) COSMIC database (Forbes *et al.*, 2011) and (B) OMIM database (Hamosh *et al.*, 2005) whose yeast orthologs are in the consensus-node NaCl signaling network

S5 Mouse phenologs associated with the yeast consensus stress-activated signaling subnetwork according to the phenolog database of Woods *et al.* (2013)

S6 Cytoscape session containing the background network, candidate network, and confidence values for nodes and edges given by the inferred subnetwork ensemble

S7 Integer program code for running subnetwork inference

S8 Cytoscape session containing consensus subnetworks, including ESR consensus subnetwork

S9 Predicted ESR bifurcation points

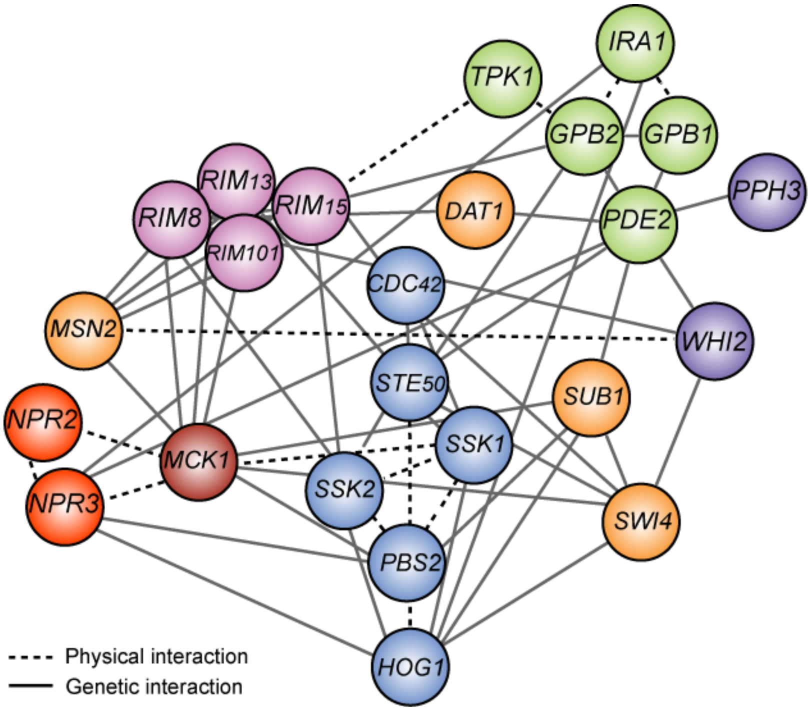

Supplementary Figure S1: Signaling proteins required for acquisition of H_2O_2 resistance. Genes required for acquisition of severe H_2O_2 resistance after 60 min treatment with 0.7M NaCl were identified in (Berry *et al.*, 2011). Shown are known signaling proteins from the selection and their physical or genetic interactions reported in the BioGRID database (Stark *et al.*, 2006). Nodes are colored according to known players in the Hog (blue), Rim (pink), Tor (dark orange), PKA (green), and Mck1 (brick) pathways. Transcription factors are colored orange, and phosphatases are colored dark purple.

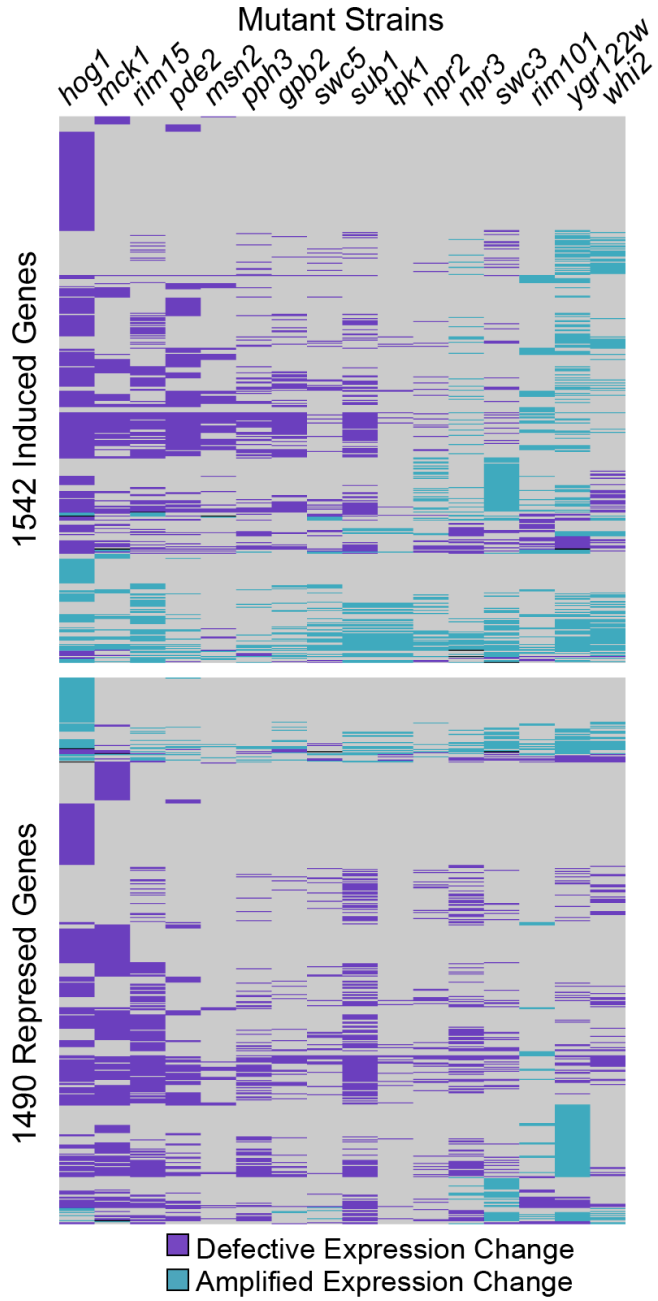

Supplementary Figure S2: Graphical representation of affected genes. Genes induced (top) or repressed (bottom) in wild type cells responding to NaCl were identified (FDR < 0.05). Genes (rows) affected in each designated mutant (columns) were identified as described in Methods. Genes with a significant defect in expression compared to wild type (i.e. a smaller fold-change) are colored in purple, and genes with a significant amplification compared to wild type (i.e. larger fold-change) are colored in cyan. Of the 5056 genes with significant NaCl-responsive changes in wild-type cells (FDR < 0.05), 3,300 genes shown here displayed a significant defect in at least one mutant. A third of the affected genes were dependent on two or more regulators. There was also significant overlap in the targets of several regulators (see also Figure 1). For example, many genes regulated by the NaCl-activated Hog1 kinase were also affected by the phosphodiesterase Pde2, which inhibits PKA signaling, and the nutrient-responsive Rim15 and Mck1 kinases not previously linked to NaCl stress.

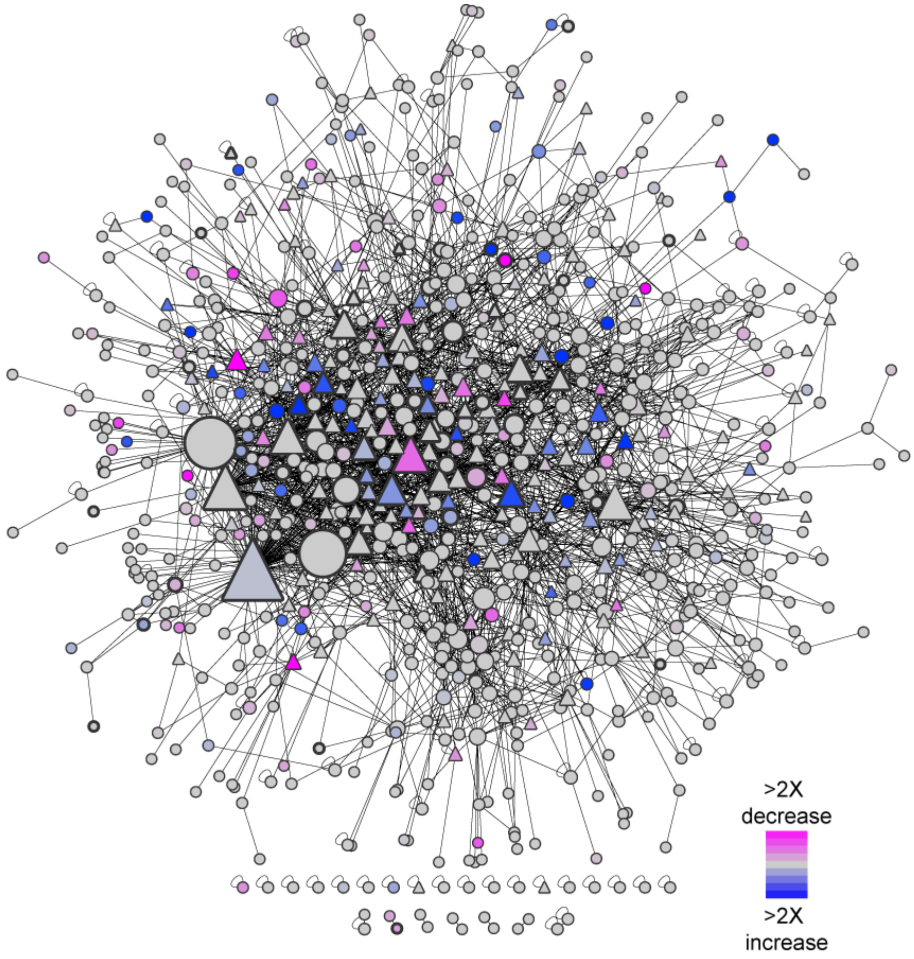

Supplementary Figure S3: Phospho-proteome network. Full caption to follow.

Caption for Supplementary Figure S3: Phospho-proteome network. Changes in the phospho-proteome were identified by quantifying phospho-peptides before and at 5 and 15 min after NaCl treatment, as described in Methods. We identified 1937 phospho-peptides mapping to 973 different proteins. Roughly 600 sites displayed a two-fold change in expression at one or both time points, roughly split between proteins with increased and decreased phosphorylation. We compared our dataset to a prior phospho-proteome analysis that identified 169 proteins with a $\geq$ two-fold change in phosphorylation upon treatment with 0.4M NaCl for 5 min and 20 min (Soufi *et al.*, 2009). Not surprisingly given the sampling challenges of phosphoproteomics and the different experimental setups, the overlap between the datasets was low (only 12 changing phospho-proteins identified by both studies). This discrepancy could be caused from the difference of culturing conditions and stress dose (0.4M in that study versus 0.7 M used here), as well as the variable peptide sampling in different mass spectrophotometer runs, a known challenge in phosphoproteomics (*e.g.*, Bodemiller *et al.* (2007)).

We used our phospho-proteomic data to build a network of the phospho-proteome, based on

previously measured protein-protein interactions. The figure shows physical interactions between phospho-proteins, where triangles represent proteins included in the inferred consensus subnetwork, and circles indicate phospho-proteins that were not included in the consensus signaling subnetwork. Node size is proportionate to degree in the phospho-proteome network, and node color is proportionate to the average fold-change in phosphorylation across both timepoints according to the color key. Nodes with more than one changing phospho-site are colored according to the site with the largest fold-change. Nodes with dark outlines indicate kinases.

The network reveals high connectivity among phospho-proteins, with many interactions corresponding to protein complexes. Two hundred (21%) of the 973 phospho-proteins were included in the final signaling network, demonstrating that these networks are partially overlapping at best. Proteins participating in the phospho-proteomic network were enriched for specific functional categories, including signal transduction, cell-cycle progression, cytokinesis/bud-site selection, and actin organization (Bonferroni-corrected $p < 0.05$), suggesting downstream effects on multiple physiological responses. Among the hubs unique to the protein network were palmitoylated plasma membrane-bound casein kinase 1 isoform Yck2 and a poorly characterized kinase, Ptk1, linked to spermine uptake. Proteins interacting with Yck2 were enriched for those involved in cell morphogenesis and linked to the cell periphery, whereas proteins linked to Ptk1 were enriched for kinases and proteins in the actin cytoskeleton. Other hubs in the phospho-protein network were Yck1, whose interacting proteins are involved in RNA transport, cell morphogenesis, and localized to the bud neck, and Atg1, a kinase regulating autophagy and vesicular trafficking and whose interacting partners were enriched for RBPs and proteins localized to the nucleolus.

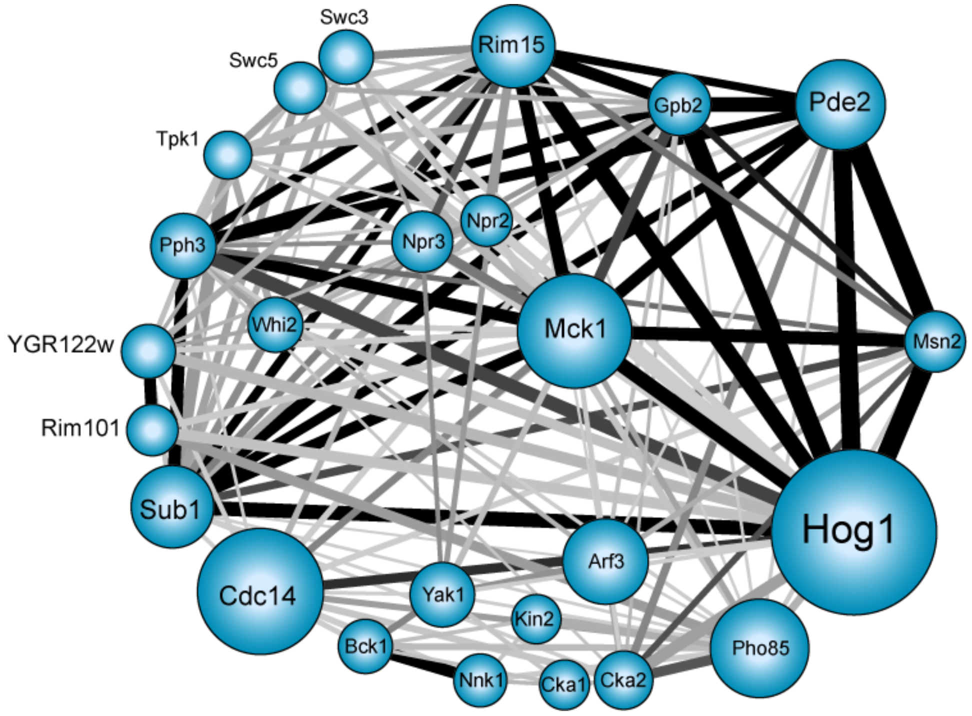

Supplementary Figure S4: Overlap in targets of all interrogated regulators. As shown in Figure 1, but including regulators profiled through validation studies. Edge thickness represents the fraction of the smaller node's targets that overlap between two nodes. Edge color is proportional to significance of the overlap, where black represents a -log(p-value) of 5 or greater. The figure represents overlap only in targets with defective NaCl-dependent expression changes (i.e. smaller change in transcript abundance compared to wild type).

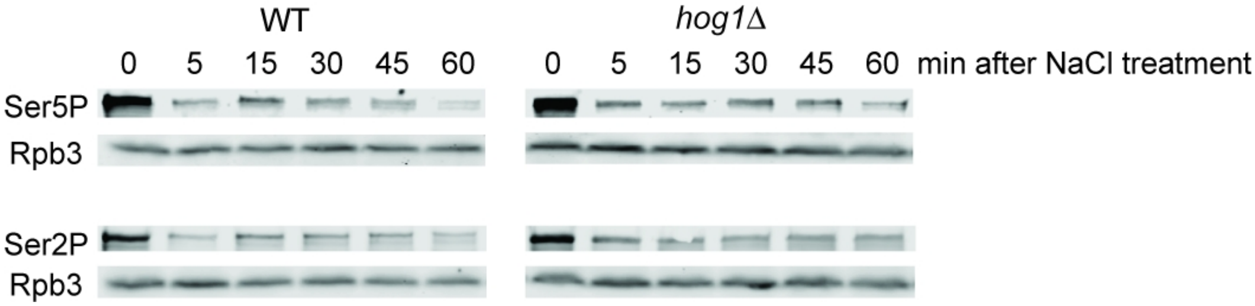

Supplementary Figure S5: Defective *in vivo* CTD phosphorylation in *hog1*Δ cells responding to NaCl. This figure shows the blots that were quantified for Figure 5D and represents reproducible biological duplicate experiments.

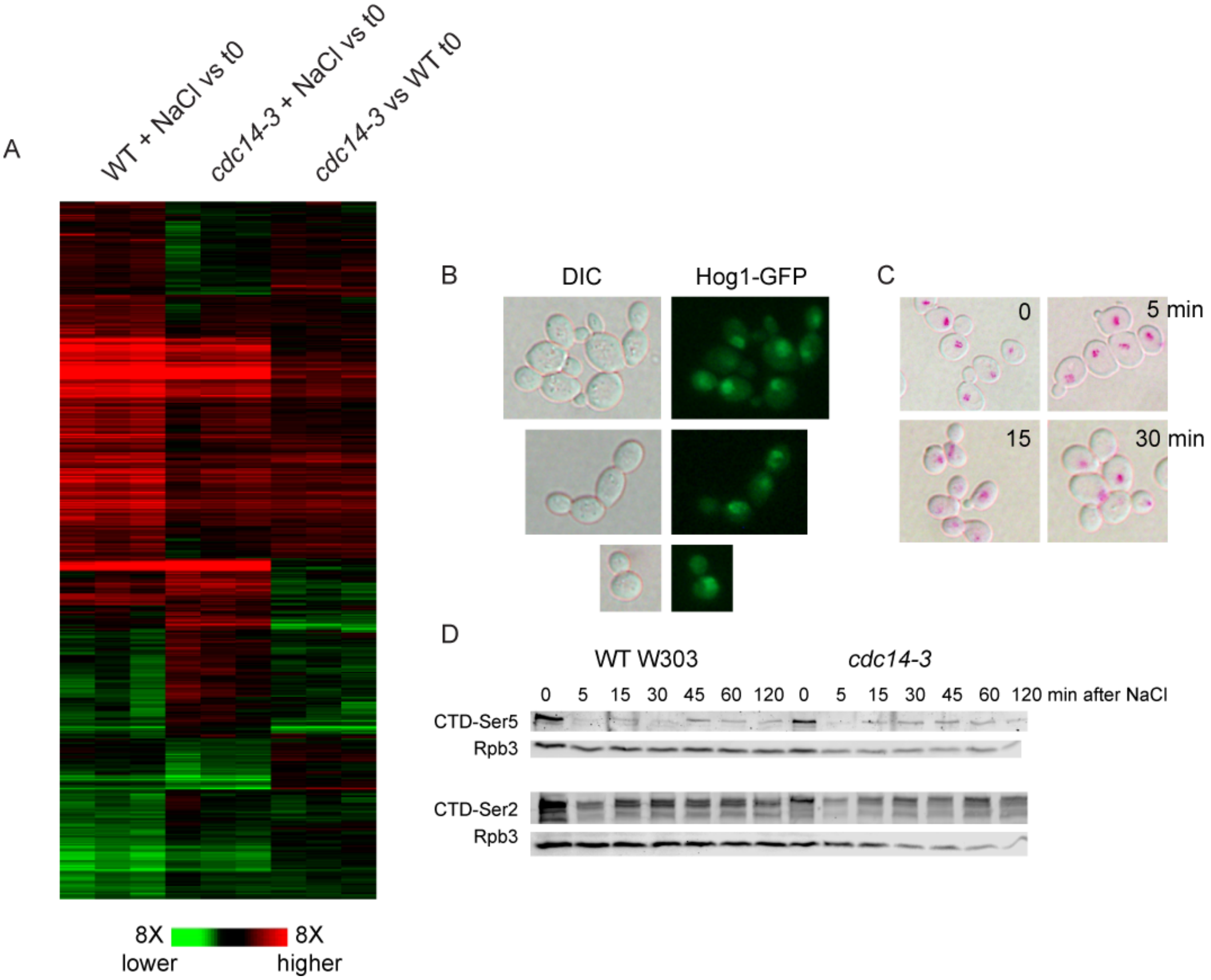

Supplementary Figure S6: Cdc14 is required for normal NaCl response.

A Expression of 940 genes (excluding cell-cycle regulated genes (Spellman *et al.*, 1998)) scored as differentially expressed in the *cdc14-3* mutant responding to NaCl ($q < 0.05$). Each experiment was done in biological triplicate.

B Examples of dumbbell-shaped wild type W303 cells with nuclear Hog1-GFP at 10 min after NaCl treatment.

C Representative pseudo-colored overlays of Cdc14-GFP cells responding to 0.7M NaCl at denoted times before (0 min) or after treatment. GFP signal is pseudocolored as magenta.

D Western blots of bulk CTD Ser5-P and Rpb3 loading control (top) and Ser2-P and Rpb3 loading control (bottom) in wild-type and *cdc14-3* cells grown at 35°C for 90 min (“0 min” NaCl treatment) and then exposed to 0.7M NaCl at 35°C for 120 min after salt treatment.

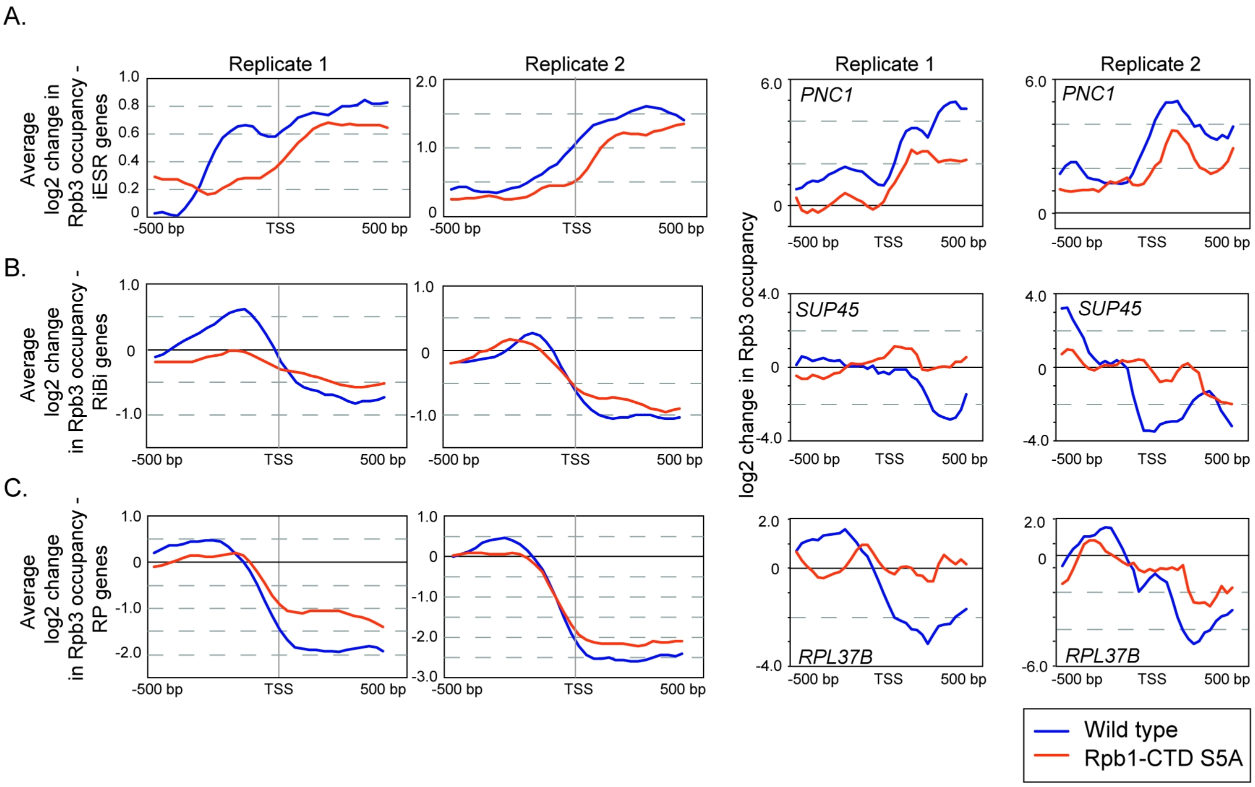

Supplementary Figure S7: Change in Rpb3 occupancy at ESR-gene promoters. The $\log_2$(fold-change) in Rpb3 occupancy is shown from -500bp to +500bp around the transcription start site (TSS) of ESR genes, for A) iESR genes, B) RiBi genes, and C) RP genes. Wild-type profiles are shown in blue and profiles from the S5A mutant are shown in orange. Data from two biological replicates is shown, averaged across all genes in the respective groups (left two panels) and for a representative gene in each group (right two panels), including iESR gene *PNC1*, RiBi gene *SUP45*, and RP gene *RPL37B*.

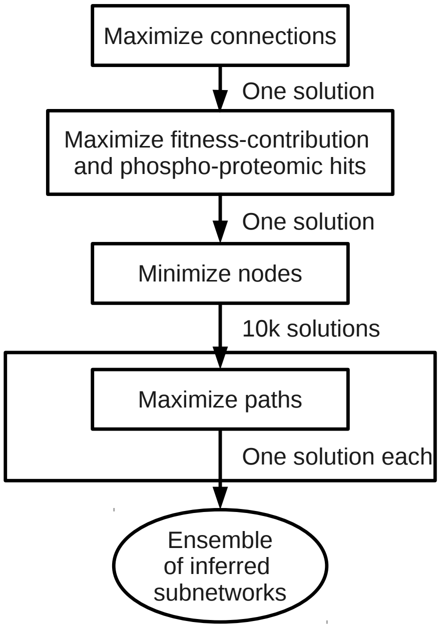

Supplementary Figure S8: Diagram of the procedure for optimization in the IP.

A

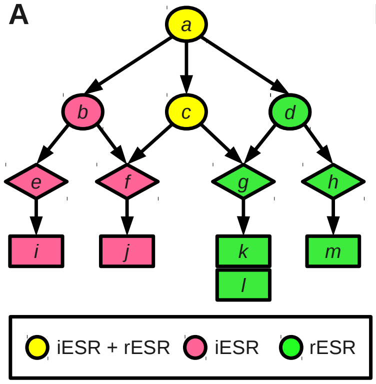

B

**Target set**	***C(T)***
T(a)	4.86
T(c)	2.76
T(b only)	0.0
T(b and c)	0.0
T(c and d)	0.0
T(d only)	0.0
T(f)	0.0
T(g)	0.0

C

n	***I(n)***	***S(n)***	***B(n)***
a	4.86	1.92	2.53
c	2.76	0.92	3.00

Supplementary Figure S9: Example of ESR bifurcation score calculation.

A Example ESR network with nodes labeled according to which ESR targets are downstream. Nodes *a* and *c* are clearly candidate bifurcation points: they are upstream of both clusters, and we can see that the paths leading out from them divide the two clusters. However, choosing and ranking possible bifurcation points in the ESR consensus subnetwork is not easy to do by visual inspection.

B Calculation of *C(T)*, count, for each candidate bifurcation point (*a* and *c*) and their outgoing partitions. At a given node, the outgoing partitions are defined by combinations of the node's children. Node *a*'s children define four outgoing partitions: ([*b* only], [*b* and *c*], [*c* and *d*], [*d* only]), and *c*'s children define two outgoing partitions ([*f*], [*g*]). This table shows all of the values of *C(·)* that must be calculated for the example network: that of the two candidate bifurcation points, *a* and *c*, and each of their outgoing partitions.

C Final scores for the candidate bifurcation points. Note that while node *a* has superior information gain *I(a)* compared to *c*, its bifurcation score *B(a)* is penalized by its high split information. Node *c* is therefore the higher-ranked bifurcation point.

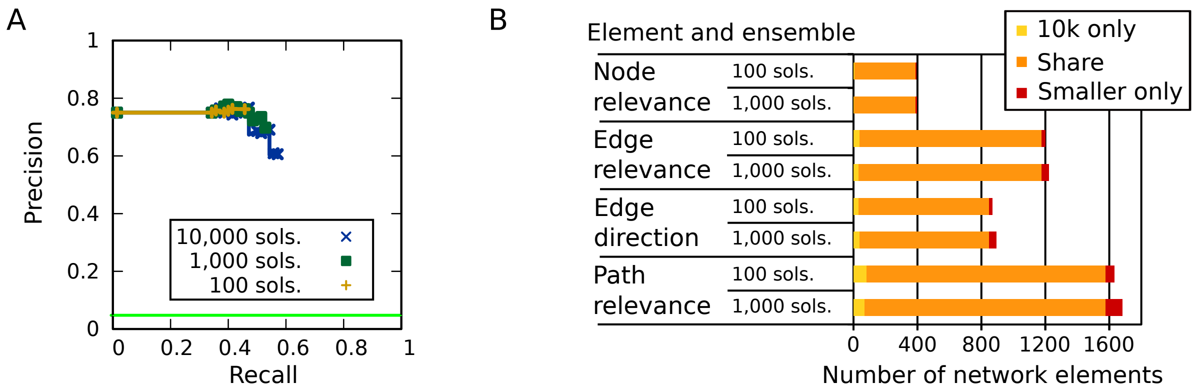

Supplementary Figure S10: IP stability analysis results.

A Precision-recall curves for inferred ensembles of three sizes. The horizontal green line in each panel indicates the precision achieved by predicting that all test cases are positive examples.

B Similarity of confidence values for different types of network elements (nodes, edges, paths) between different sizes of ensembles. Each bar represents the total number of elements in the union of the complete, 10,000-solution ensemble with a smaller ensemble. The central portion of the bar, “Shared”, represents the number of elements that are in the intersection of both ensembles. The end caps, labeled “10k only” and “Smaller only”, represent the number of elements unique to each ensemble. The longer the center is relative to the ends, the more similar are the two ensembles in their predictions.

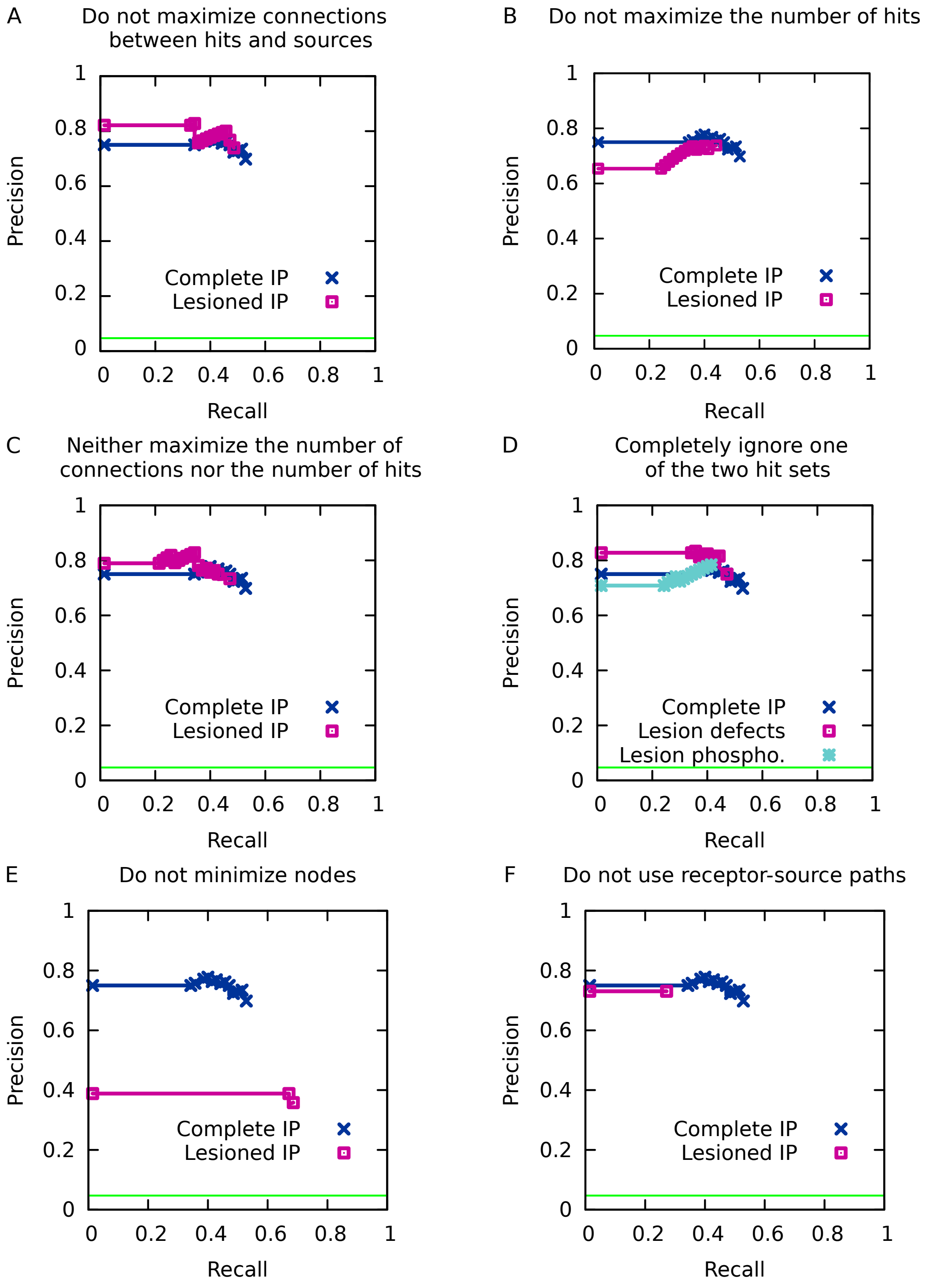

Supplementary Figure S11: Precision-recall curves for the lesioned IP. The horizontal green line in each panel indicates the precision achieved by predicting that all test cases are positive examples. The lesioned components are:

A maximization of connections,
B maximization of hits,
C maximization of both connections and hits,
D an entire set of experimental node labels (fitness-contributions or phospho-proteins),
E minimization of nodes,
F inclusion of receptor-source pairs curated from literature.

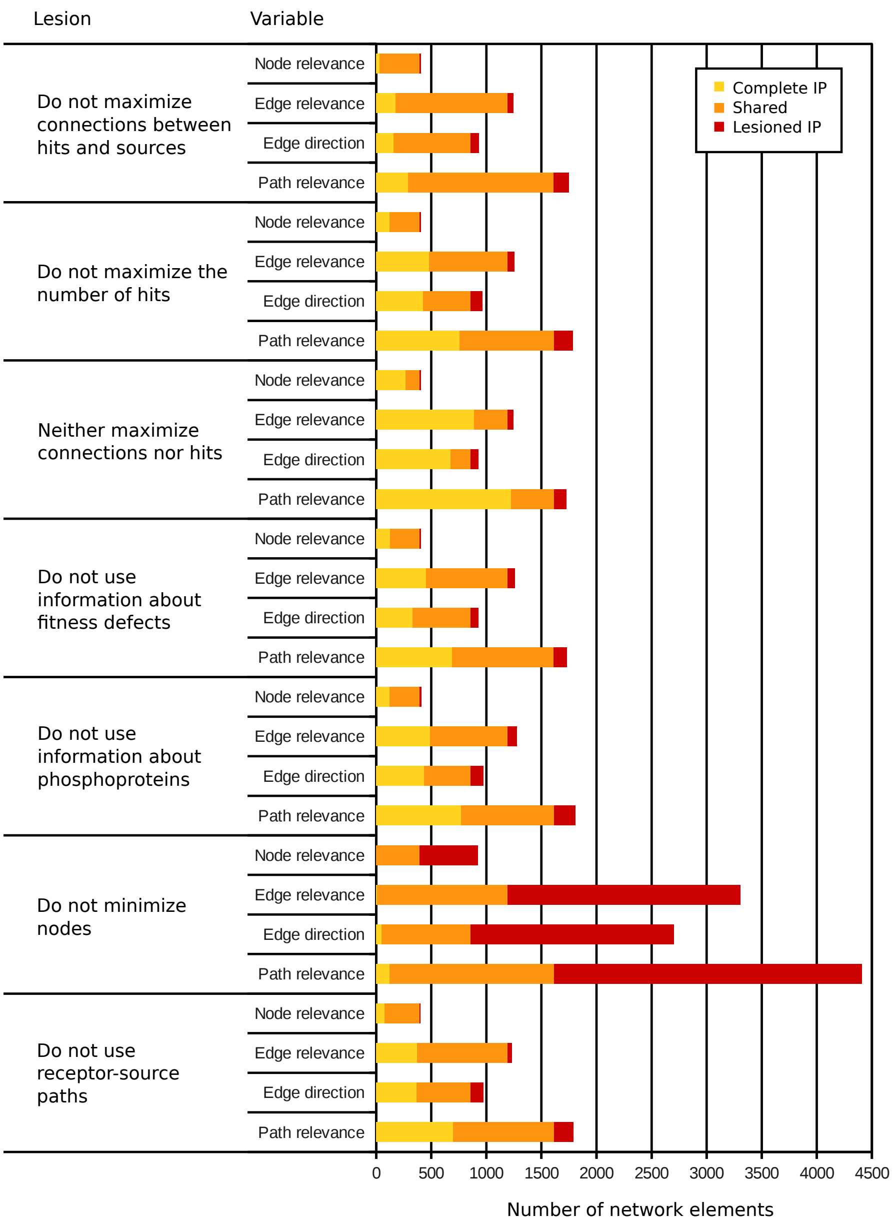

Supplementary Figure S12: Similarity of ensembles inferred by the lesioned IPs. Comparison of the complete ensemble to the lesioned ensembles on the basis of the confidence values given to each type of network element (nodes, edges, paths). Each bar represents the total number of elements in the union of the complete ensemble with a lesioned ensemble. The central portion of the bar, “Shared”, represents the number of elements that are in the intersection of both ensembles. The end caps, labeled “Complete IP” and “Lesioned IP”, represent the number of elements unique to each ensemble. The longer the center is relative to the ends, the more similar are the two ensembles in their predictions.

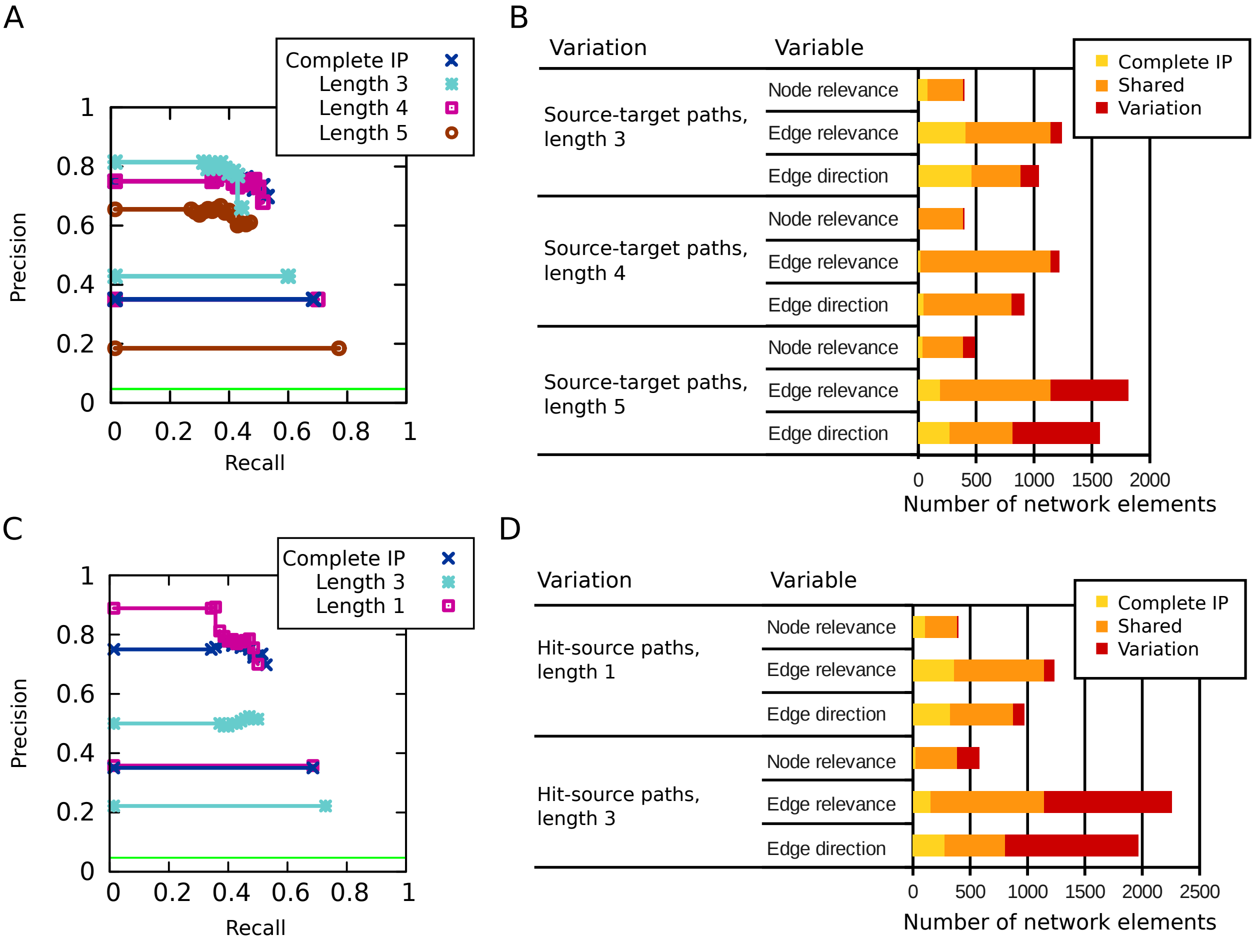

Supplementary Figure S13: Precision-recall curves and stability results derived from changing the length of candidate paths. For each variation, we show two PR curves in the same color. The top curve represents the inferred ensemble, while the bottom curve represents the performance of candidate paths provided as input to the inference m. The horizontal green line in PR plot indicates the precision achieved by predicting that all test cases are positive examples. In the stability analysis panel, each bar represents the total number of elements in the union of the complete ensemble with a lesioned ensemble. The central portion of the bar, “Shared”, represents the number of elements that are in the intersection of both ensembles. The end caps, labeled “Complete IP” and “Variation”, represent the number of elements unique to each ensemble. The longer the center is relative to the ends, the more similar are the two ensembles in their predictions. In contrast to previous stability analyses, we do not compare the ensembles based on path relevance, as the input sets of paths are different.

A Precision-recall analysis for varying source-target path length

B Stability analysis for varying source-target path length

C Precision-recall analysis for varying hit-source path length

D Stability analysis for varying hit-source path length

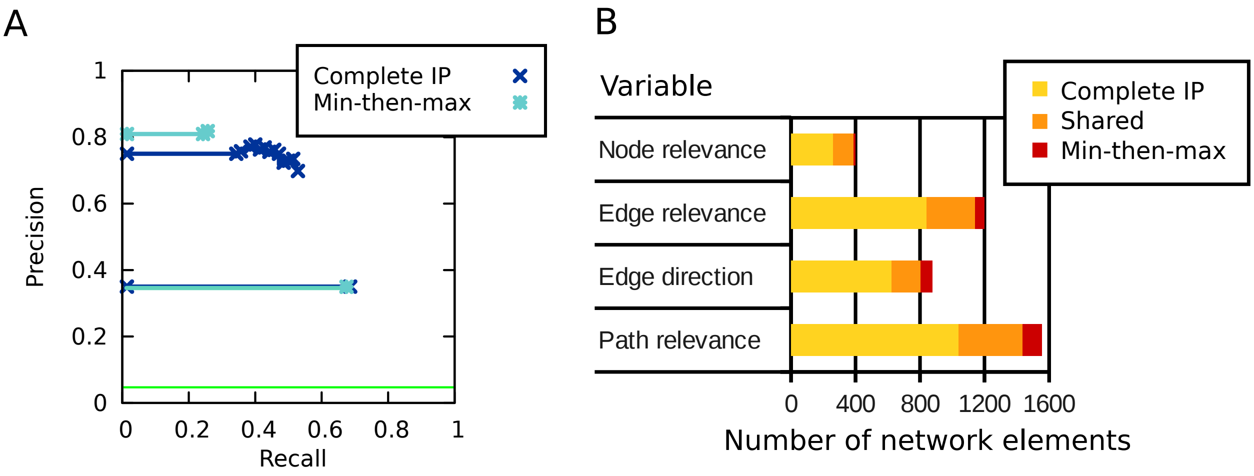

Supplementary Figure S14: Precision-recall curves and stability results derived from reversing the order in which the objective function components are solved.

A Precision-recall analysis. For each ensemble, two PR curves are shown, one for each variation on the objective function procedure. The top curve represents the performance of the inferred subnetwork, while the bottom curve represents the performance of input candidate paths. The curves for candidate paths are identical, as the input was the same for both versions. The horizontal green line in each panel indicates the precision achieved by predicting that all test cases are positive examples.

B Stability analysis. Each bar represents the total number of elements in the union of the complete ensemble with a lesioned ensemble. The central portion of the bar, “Shared”, represents the number of elements that are in the intersection of both ensembles. The end caps, labeled “Complete IP” and “Min-then-max”, represent the number of elements unique to each ensemble. The longer the center is relative to the ends, the more similar are the two ensembles in their predictions.

Supplementary Table S1: Validation of predicted regulators.
* = data from Gitter *et al.* (2013). ^ T0 = unstressed mutant. + = data from Alejandro-Osorio *et al.* (2009).
NA = no targets predicted, by nature of the node inclusion.

Predicted regulators	Defective NaCl transcriptome response	Significant overlap in measured vs. predicted targets (p-value)	Number of in-path validated nodes	Number of scorable in-path nodes	Percent validated in-path nodes
Arf3	Yes	Yes (p =6e-5)	2	2	100%
Bck1	Yes	NA	2	4	50%
Bem1*	Yes	NA	1	4	25%
Cdc14	Yes	Yes (2e-14)	14	18	78%
Cka2	Yes	Yes (3e-3)	7	10	70%
Ckb1/2	Yes	No (1); overlap with Cka2 targets (p =3e-12)	8	21	38%
Gal11*	Yes	Yes (p =2e-4)	5	10	50%
Kin2^	Yes	No (p =0.57); marginal overlap with T0-affected ($p = 0.005$)	3	17	18%
Nkk1	Yes	No (p =1)			
Pho85	Yes	Yes (p =5e-8)	14	21	67%
Rpd3+	Yes	NA			
Scd6	No				
Tpk2*	Yes	Yes (p =5e-20)	10	24	42%
Yak1	Yes	Yes (p =1e-10)	5	10	50%

Supplementary Table S2: Provenance of interactions in the background network. Notes about database extraction procedures are given in italicized text.

Interaction	Source	Directed	Count
	Protein-protein interactions		
General protein-protein	Stark *et al.* (2006) (BioGRID)	Both	17,306
	Interactions supported by at least two categories of experimental methods; treated phosphorylation edges separately; downloaded 3/2011		
PPIs involving kinases and phosphatases	Breitkreutz *et al.* (2010) (YeastKinome.org)	No	989
	Fasolo *et al.* (2011)	No	1,028
	Sharifpoor *et al.* (2011) (Yeast KID)	No	138
	Interactions without evidence of direct phosphorylation and annotated with p<0.01; downloaded 9/2012		
Kinase-substrate interactions	Sharifpoor *et al.* (2011)	Yes	414
	Annotated with p < 0.01 and one of the following evidence codes: "(LTP in vitro kinase assays OR In vitro phosphorylation site mapping (Mass Spec, Phospho-specific antibodies by Western, in vitro site-directed mutagenesis) OR In vivo site-directed mutagenesis in substrate showing same biological consequence as the kinase delete OR Reduction in phospho-peptide in vivo by mass-spec OR In vivo phosphorylation site mapping using phospho-specific antibodies (Western blot) or by phospho-peptide mapping)"; downloaded 9/2012		
	Ptacek *et al.* (2005); Stark *et al.* (2006)	Yes	5,315
	All interactions from Ptacek et al. *(2005), plus low-throughput phosphorylation/dephosphorylation interactions from BioGRID, March 2011*		
Manually curated	This work	Both	17
	Hand-constructed after inspection of neighborhoods of interrogated sources		

Continued on next page

Interaction	Source	Directed	Count
	Protein-nucleic acid interactions		
Protein-DNA	Abdulrehman *et al.* (2011); Everett *et al.* (2009); Guelzim *et al.* (2002); MacIsaac *et al.* (2006); Venters *et al.* (2011)	Yes	259,565
Osmotic-stress specific, protein-DNA	Huebert *et al.* (2012)	Yes	1,225
Salt-stress specific protein-DNA	Ni *et al.* (2009)	Yes	2,144
Protein-RNA	Hogan *et al.* (2008); Scherrer *et al.* (2010); Tsvetanova *et al.* (2010)	Yes	17,868
	Other interaction types		
Between metabolic enzymes	Heavner *et al.* (2012)	Both	1,153
	Created binary interactions from enzymes reported to catalyze adjacent reactions; reported as "directed" if the reaction was annotated as "irreversible"		
Complex membership	Heavner *et al.* (2012); Pu *et al.* (2009)	Yes	2,183
	Directed interactions from protein to complex		
Inferred complex-complex interactions	Heavner *et al.* (2012); Pu *et al.* (2009), Stark *et al.* (2006)	No	22
	Interaction between two complexes inferred if >50% possible protein pairs have interactions in BioGRID		
Inferred complex-protein interactions	Heavner *et al.* (2012); Pu *et al.* (2009), Stark *et al.* (2006)	No	1,128
	Interaction between complex and protein inferred if >50% possible protein pairs have interactions in BioGRID		

Supplementary Table S3: Sets of network elements that are provided as input to the method.

Network elements	Set	Description
Nodes	$\mathcal{N}$	All nodes
	$\mathcal{N}^S$	Source nodes
	$\mathcal{N}^R$	Receptor nodes
	$\mathcal{N}^F$	Fitness-contribution hits
	$\mathcal{N}^P$	Phospho-proteomic hits
	$\mathcal{N}^T$	Target nodes
	$\mathcal{N}^T(s)$	Dysregulated targets of source s
	$\mathcal{N}^S(r)$	Sources that are downstream of receptor r
	$\mathcal{N}(e)$	Nodes in edge e
Edges	$\mathcal{E}$	All edges
	$\mathcal{E}^D$	Directed edges
	$\mathcal{E}^U$	Undirected edges
	$\mathcal{E}(n)$	Edges that touch node n
Paths	$\mathcal{P}$	All paths
	$\mathcal{P}^{ST}$	Source-target paths
	$\mathcal{P}^{ST}(s,t)$	Source-target paths between source s and target t
	$\mathcal{P}^{RS}$	Receptor-source paths
	$\mathcal{P}^{RS}(r,s)$	Receptor-source paths between receptor r and source s
	$\mathcal{P}^{FS}$	fitness-contribution hit-source paths
	$\mathcal{P}^{FS}(f,s)$	fitness-contribution hit-source paths between hit f and source s
	$\mathcal{P}^{SS}$	Source-source paths
	$\mathcal{P}^{SS}(s_i,s_j)$	Paths between source s_i and source s_j

Supplementary Table S4: Integer program variables. Binary variables represent the status of nodes, edges, paths, and pairs in the network.

Network elements	Variable	Interpretation	Values
Paths p	σ_p	Relevant	no=0, yes=1
Edges e	x_e	Relevant	no=0, yes=1
	d_e	Direction	back=0, forward=1
Nodes n	y_n	Relevant	no=0, yes=1
Pairs (n_i, n_j)	c_{ij}	Connected	no=0, yes=1

Supplementary Table S5: Coverage of each source's targets and candidate TF/RBPs by the candidate paths. 'Prop.' columns give the proportion of targets covered by candidate paths. Sources marked with the message 'Self' are sources that are themselves TFs or RBPs; for these sources, all targets were covered by the addition of inferred regulatory interactions.

	Targets			Candidate TF/RBPs		
Source	Covered	Total	Prop.	Covered	Total	Prop.
Gpb2	174	234	0.74	12	17	0.71
Hog1	1843	1912	0.96	41	44	0.93
Mck1	861	886	0.97	48	52	0.92
Npr2	71	131	0.54	9	12	0.75
Npr3	218	267	0.82	16	28	0.57
Pde2	526	573	0.92	26	41	0.63
Pph3	209	247	0.85	12	22	0.55
Rim15	513	530	0.97	36	41	0.88
Sub1	422	515	0.82	15	19	0.79
Tpk1	53	124	0.43	6	10	0.6
Whi2	167	296	0.56	8	9	0.89
Dot6/Tod6	–	259	–	–	Self	–
Msn2	–	209	–	–	Self	–
Rim101	–	302	–	–	Self	–
Swc3	–	365	–	–	Self	–
Swc5	–	138	–	–	Self	–

Supplementary Table S6: Top 15 consensus nodes ranked by degree.

Name	ORF	Degree	Annotation
Tpk1	YJL164C	69	Source, kinase
Mck1	YNL307C	44	Source, kinase
Hog1	YLR113W	42	Source, kinase
Pho85	YPL031C	31	Kinase
Cdc28	YBR160W	28	Kinase
Rpb1	YDL140C	27	RNA Pol II core subunit
Kin2	YLR096W	24	Kinase
Tpk2	YPL203W	24	Source, kinase
Cdc14	YFR028C	23	Kinase
Rad53	YPL153C	23	Kinase
Ubi4	YLL039C	23	Ubiquitin
Smt3	YDR510W	21	SUMO
Bmh1	YER177W	20	14-3-3 scaffold
Hrr25	YPL204W	20	CTD-binding kinase
Tpk3	YKL166C	19	Kinase

Supplementary Table S7: Enrichment analysis results. The ‘Prop.’ columns give the proportion of each subnetwork that is in the relevant gene or interaction set; for the ‘Permutations’ row, this value is the average over all 1,000 permutations. The ‘p-value’ columns are calculated by a comparison to the consensus subnetwork (derived from the 10,000-solution ensemble). Asterisks (*) indicate a result that is significant at $p < 0.05$. For comparisons to the candidate and background networks, p-values are calculated using the hypergeometric test. For comparisons to the permuted ensembles, p-values are calculated as the fraction of permutations having an equal or greater proportion of relevant genes (or lower propoerion of likely negatives) compared to the consensus subnetwork.

	Relevant gene sets											
	True positives		Likely negatives		Kinases and phosphatases		General stress proteins		Essential genes		Genetic interactions	
Subnetwork	Prop.	p-value	Prop.	p-value	Prop.	p-value	Prop.	p-value	Prop.	p-value	Prop.	p-value
Consensus nodes	0.156		0.050		0.200		0.269		0.294		0.051	
Candidate network	0.065	2e-6 *	0.121	2e-4 *	0.124	0.001 *	0.145	2e-6 *	0.319	0.810	0.033	≈ 0 *
Background network	0.015	5e-20 *	0.302	6e-17 *	0.032	8e-18 *	0.050	5e-21 *	0.194	0.001 *	0.008	≈ 0 *
Permutations	0.072	0.002 *	0.133	0.007 *	0.183	0.309	0.185	0.007 *	0.348	0.899	0.035	0.003 *

Supplementary Table S8: Enrichment analysis of lesioned IPs. 'Prop' columns show the proportion of each lesioned or complete consensus subnetwork that is composed of the relevant gene set being tested. P-values are calculated by a two-tailed z-test of proportions comparing the complete consensus subnetwork (derived from the 1,000 solution ensemble) to the consensus subnetwork inferred using a different path length (also derived from 1,000-solution ensembles). Asterisks (*) indicate significance at $p < 0.05$. For significant results, bolded proportions and p-values indicate comparisons in which the original, complete IP has a higher proportion of relevant genes/interactions (or lower proportion of likely negatives). Italicized proportions and p-values indicate the opposite.

	Relevant gene sets											
	True positives		Likely negatives		Kinases and phosphatases		General stress proteins		Essential genes		Genetic interactions	
IP Version	Prop.	p-val.	Prop.	p-val.	Prop.	p-val.	Prop.	p-val.	Prop.	p-val.	Prop.	p-val.
Complete IP (1k sols.)	0.168		0.054		0.197		0.257		0.293		0.052	
Do not maximize connections	0.179	0.801	0.057	0.901	0.200	0.958	0.250	0.881	0.279	0.775	**0.047**	≈**0** *
Do not maximize hits	0.131	0.380	0.066	0.664	0.182	0.738	0.255	0.968	0.263	0.554	*0.061*	≈*0* *
Neither maximize connections nor hits	0.183	0.757	0.043	0.700	0.172	0.613	0.237	0.709	0.247	0.426	*0.071*	≈*0* *
Do not use fitness-defects	0.202	0.454	0.047	0.774	0.202	0.933	0.256	0.974	0.310	0.757	0.050	0.242
Do not use phospho-hits	0.133	0.409	0.052	0.937	0.193	0.913	0.252	0.911	0.259	0.510	*0.060*	≈*0* *
Do not minimize nodes	**0.067**	**0.000 ***	**0.113**	**0.024 ***	**0.121**	**0.009 ***	**0.147**	**0.001 ***	0.315	0.591	**0.033**	≈**0 ***
Do not use receptor-source paths	0.133	0.394	0.049	0.845	0.182	0.724	0.273	0.762	0.273	0.687	0.052	0.553

Supplementary Table S9: Enrichment analysis of subnetworks inferred from varied candidate path lengths. 'Prop' columns show the proportion of each consensus subnetwork that is composed of the relevant gene set being tested. P-values are calculated by a two-tailed z-test of proportions comparing the complete consensus subnetwork (derived from the 1,000 solution ensemble) to the consensus subnetwork inferred using a different path length (also derived from 1,000-solution ensembles). Asterisks (*) indicate significance at $p < 0.05$. For significant results, bolded proportions and p-values indicate comparisons in which the original, complete IP has a higher proportion of relevant genes/interactions (or lower proportion of likely negatives). Italicized proportions and p-values indicate the opposite.

	Relevant gene sets											
	True positives		Likely negatives		Kinases and phosphatases		General stress proteins		Essential genes		Genetic interactions	
IP Version	Prop.	p-value	Prop.	p-value	Prop.	p-value	Prop.	p-value	Prop.	p-value	Prop.	p-value
Complete IP (1k sols.)	0.168		0.054		0.197		0.257		0.293		0.052	
Source-target paths, length 3	0.176	0.857	0.046	0.751	0.221	0.616	0.260	0.968	0.328	0.518	*0.057*	$\approx$ *0* *
Source-target paths, length 4	0.168	1.000	0.048	0.803	0.204	0.891	0.258	1.000	0.287	0.904	0.052	0.504
Source-target paths, length 5	0.124	0.260	0.071	0.517	0.195	0.960	0.225	0.484	0.343	0.327	**0.044**	$\approx$ **0 ***
Source-hit paths, length 1	0.191	0.604	0.038	0.524	0.214	0.731	0.260	0.970	0.282	0.836	*0.061*	$\approx$ *0* *
Source-hit paths, length 3	**0.100**	**0.039** *	0.107	0.055	0.184	0.724	0.207	0.223	*0.387*	*0.048* *	**0.041**	$\approx$ **0 ***

Supplementary Table S10: Enrichment analysis of reordered IPs. ‘Prop’ columns show the proportion of each consensus subnetwork that is composed of the relevant gene set being tested. P-values are calculated from a two-tailed z-test of proportions comparing the complete consensus subnetwork (derived from 1,000 solutions) to the consensus subnetwork inferred using a different ordering of the objective function procedure (also derived from 1,000 solutions). Asterisks (*) indicate significance at $p < 0.05$. For the significant result, the italicized proportion (and p-value) indicates a comparison in which the original, complete IP has a lower proportion of relevant genetic interactions.

	Relevant gene sets											
	True positives		Likely negatives		Kinases and phosphatases		General stress proteins		Essential genes		Genetic interactions	
IP Version	Prop.	p-value	Prop.	p-value	Prop.	p-value	Prop.	p-value	Prop.	p-value	Prop.	p-value
Complete IP (1k sols.)	0.168		0.054		0.197		0.257		0.293		0.052	
Min-then-max	0.181	0.787	0.043	0.686	0.170	0.586	0.255	0.969	0.245	0.398	*0.076*	*≈ 0 **
