# Supplementary figures and images for: Pathway connectivity and signaling coordination in the yeast stress-activated signaling network
Source: Mol Syst Biol. 2014 Nov 19;10(11):759. doi: 10.15252/msb.20145120 (PMC4299600; doi:10.15252/msb.20145120)

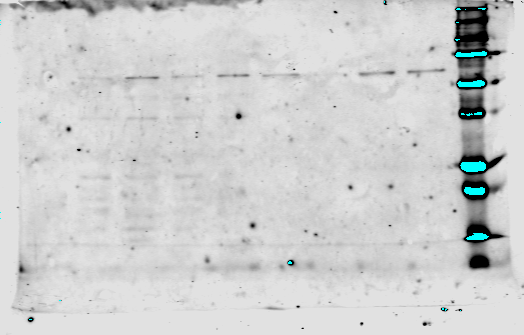

Supplement: Supplementary file 12 — Source Data for Figure 6C, D [file msb0010-0759-sd12.zip › Source_Data_Fig_6C_and_D/Cka1_PullDown/Cka1-Pulldown_antiCdc14.tif]

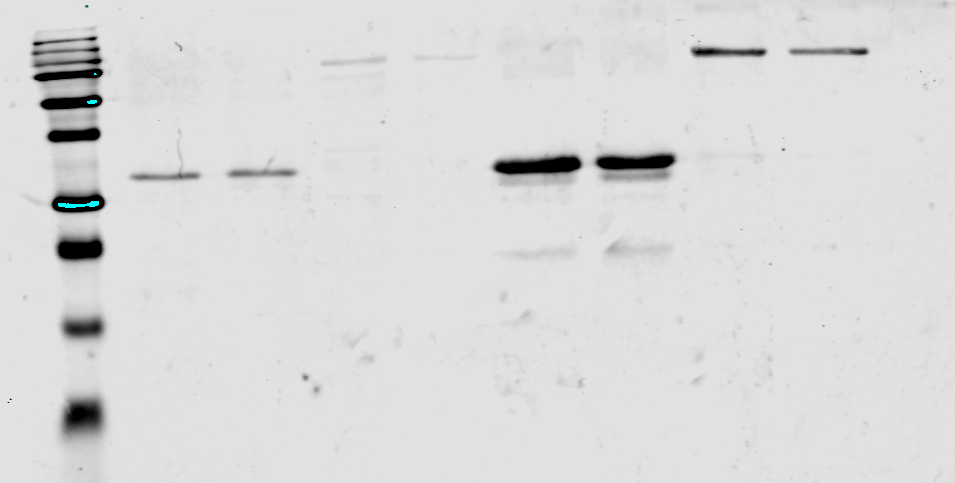

Supplement: Supplementary file 12 — Source Data for Figure 6C, D [file msb0010-0759-sd12.zip › Source_Data_Fig_6C_and_D/Cka1_PullDown/Cka1-Pulldown_antiGST.tif]

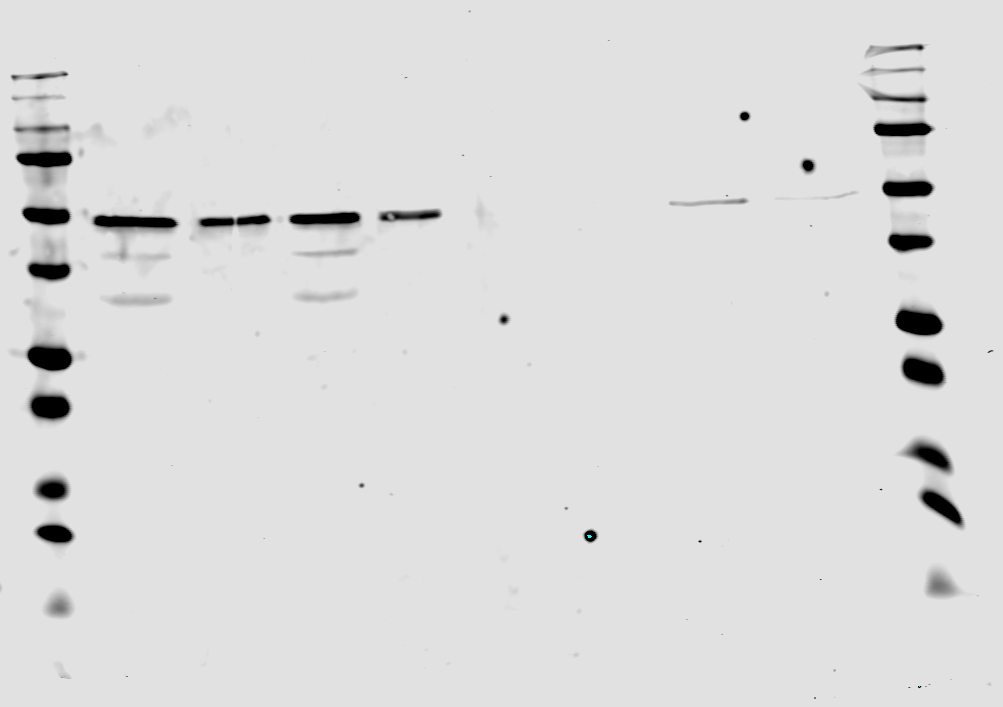

Supplement: Supplementary file 12 — Source Data for Figure 6C, D [file msb0010-0759-sd12.zip › Source_Data_Fig_6C_and_D/Cka1_PullDown/Cka1-Pulldown_antiHog1.tif]

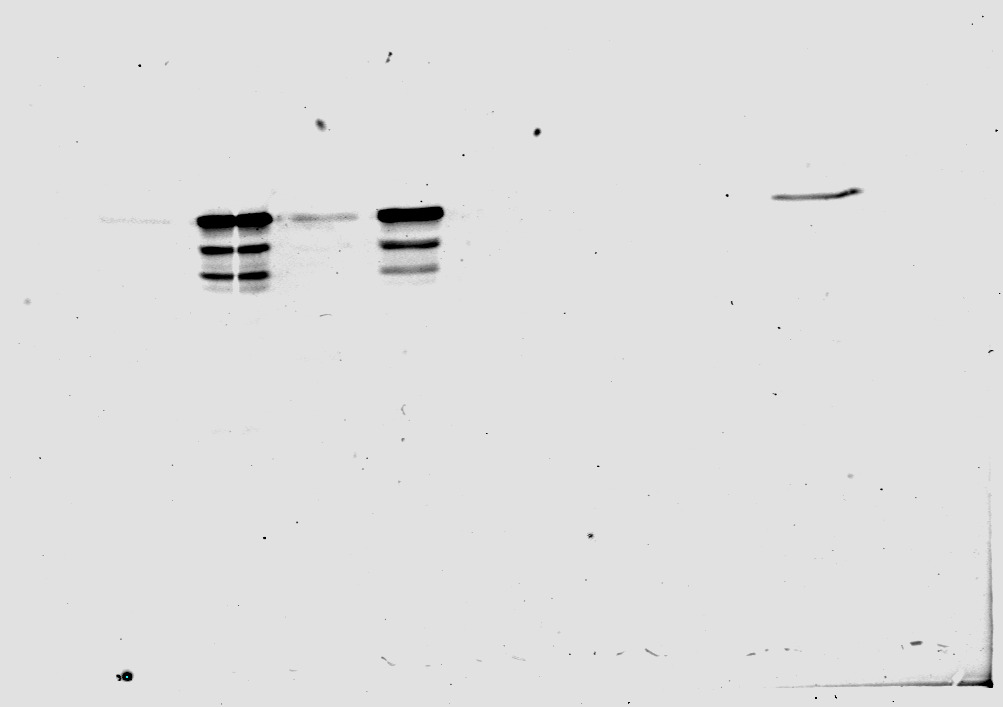

Supplement: Supplementary file 12 — Source Data for Figure 6C, D [file msb0010-0759-sd12.zip › Source_Data_Fig_6C_and_D/Cka1_PullDown/Cka1-Pulldown_antiPhosphoHog1.tif]

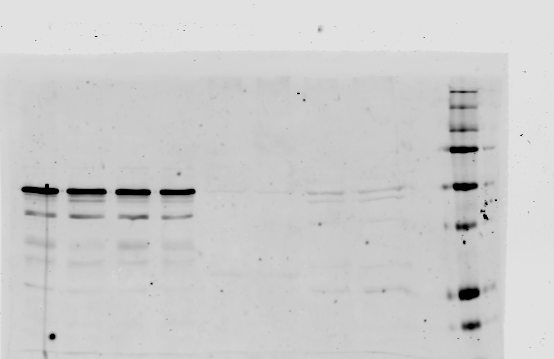

Supplement: Supplementary file 12 — Source Data for Figure 6C, D [file msb0010-0759-sd12.zip › Source_Data_Fig_6C_and_D/Cka2_PullDown/072613 CI9_2 Cka2 700_35 hog1_2.tif]

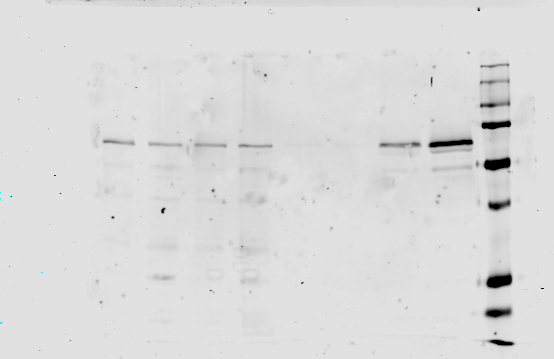

Supplement: Supplementary file 12 — Source Data for Figure 6C, D [file msb0010-0759-sd12.zip › Source_Data_Fig_6C_and_D/Cka2_PullDown/072613 CI9_2 Cka2 700_4 cdc14.tif]

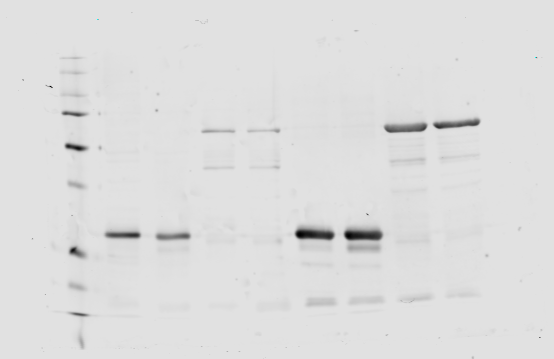

Supplement: Supplementary file 12 — Source Data for Figure 6C, D [file msb0010-0759-sd12.zip › Source_Data_Fig_6C_and_D/Cka2_PullDown/072613 CI9_2 Cka2 700_4 gst.tif]

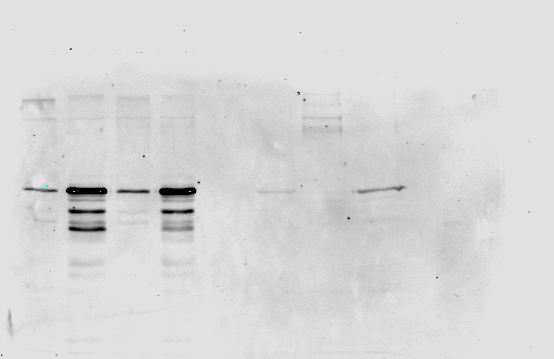

Supplement: Supplementary file 12 — Source Data for Figure 6C, D [file msb0010-0759-sd12.zip › Source_Data_Fig_6C_and_D/Cka2_PullDown/072613 CI9_2 Cka2 800_7 p-hog_2.tif]
